# The tryptophan-metabolizing enzyme indoleamine 2,3-dioxygenase 1 regulates polycystic kidney disease progression

**DOI:** 10.1172/jci.insight.154773

**Published:** 2023-01-10

**Authors:** Dustin T. Nguyen, Emily K. Kleczko, Nidhi Dwivedi, Marie-Louise T. Monaghan, Berenice Y. Gitomer, Michel B. Chonchol, Eric T. Clambey, Raphael A. Nemenoff, Jelena Klawitter, Katharina Hopp

**Affiliations:** 1 Department of Medicine, Division of Renal Diseases and Hypertension,; 2 Consortium for Fibrosis Research and Translation, and; 3 Department of Anesthesiology, University of Colorado Anschutz Medical Campus, Aurora, Colorado, USA.

**Keywords:** Nephrology, Amino acid metabolism, Cellular immune response, Monogenic diseases

## Abstract

Autosomal dominant polycystic kidney disease (ADPKD), the most common monogenic nephropathy, is characterized by phenotypic variability that exceeds genic effects. Dysregulated metabolism and immune cell function are key disease modifiers. The tryptophan metabolites, kynurenines, produced through indoleamine 2,3-dioxygenase 1 (IDO1), are known immunomodulators. Here, we study the role of tryptophan metabolism in PKD using an orthologous disease model (C57BL/6J *Pkd1*^RC/RC^). We found elevated kynurenine and IDO1 levels in *Pkd1*^RC/RC^ kidneys versus wild type. Further, IDO1 levels were increased in ADPKD cell lines. Genetic *Ido1* loss in *Pkd1*^RC/RC^ animals resulted in reduced PKD severity, as measured by cystic index and percentage kidney weight normalized to body weight. Consistent with an immunomodulatory role of kynurenines, *Pkd1*^RC/RC^;*Ido1^–/–^* mice presented with significant changes in the cystic immune microenvironment (CME) versus controls. Kidney macrophage numbers decreased and CD8^+^ T cell numbers increased, both known PKD modulators. Also, pharmacological IDO1 inhibition in *Pkd1*^RC/RC^ mice and kidney-specific *Pkd2*-knockout mice with rapidly progressive PKD resulted in less severe PKD versus controls, with changes in the CME similar to those in the genetic model. Our data suggest that tryptophan metabolism is dysregulated in ADPKD and that its inhibition results in changes to the CME and slows disease progression, making IDO1 a therapeutic target for ADPKD.

## Graphical abstract

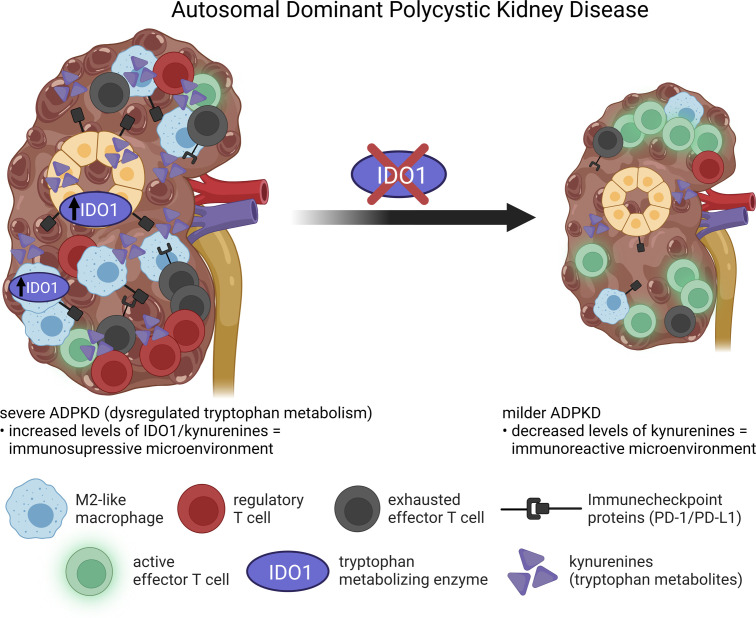

## Introduction

Autosomal dominant polycystic kidney disease (ADPKD) is the most common, life-threatening genetic kidney disease (1, 2). It is characterized by progressive kidney cyst growth that leads to organ failure and accounts for 5%–10% of end-stage kidney disease cases worldwide (3, 4). Tolvaptan, a vasopressin receptor antagonist, is the only FDA-approved therapy for ADPKD, which slows cyst growth but also impairs quality of life, underscoring the need for alternative therapies (5, 6). ADPKD is predominantly caused by mutations to *PKD1* or *PKD2*, but manifests with significant inter- and intrafamilial phenotypic variability that exceeds genic or allelic effects (7–9). Disease variability has been linked to genetic modifiers, kidney injury, and environmental/lifestyle factors (7, 10–12).

Accumulating evidence suggests that metabolic reprogramming is a key feature of ADPKD (13–15). In the HALT-PKD Study A population, overweight/obesity were shown to be strong independent risk factors of total kidney volume increase and estimated glomerular filtration rate (eGFR) decline (10). Correlatively, preclinical studies in PKD models have shown that mild-to-moderate caloric restriction, time-restricted feeding, or ketogenic diet ameliorates kidney cyst growth (16–18). Indeed, targeting specific metabolic pathways found to be dysregulated in PKD such as glycolysis, fatty acid oxidation, and arginine, glutamine, and methionine metabolism alleviated cystic kidney disease in PKD models (19–24).

Data from our group and others suggest that tryptophan metabolism may also play a role in ADPKD progression (25, 26). Tryptophan is catabolized to kynurenine via tryptophan 2,3-dioxygenase (TDO) or indoleamine 2,3-dioxygnease (IDO1, IDO2; Supplemental Figure 1; supplemental material available online with this article; https://doi.org/10.1172/jci.insight.154773DS1). While TDO is predominantly expressed in the liver, IDO1 and -2 are expressed in kidney epithelial and immune cells, with IDO1 having higher catalytic activity for tryptophan (27–29). Kynurenine and/or its metabolite, kynurenic acid, are known drivers of oxidative stress, dysregulated calcium homeostasis, and mitochondrial dysfunction (30–32). As such, they are considered uremic toxins; their serum levels correlate with chronic kidney disease (CKD) severity (33). Interestingly, serum metabolomics of participants of the Modification of Diet in Renal Disease Study showed that levels of kynurenic acid were selectively elevated in ADPKD patients compared with other CKD patients. Also, we recently published that ADPKD patients have higher plasma kynurenine concentrations compared with healthy individuals and levels further increased with disease progression (25, 26).

In cancer, a disease with multiple parallels to PKD, plasma kynurenine levels and tumor expression of IDO1 negatively correlate with cancer survival and clinical outcome (34–38). Elevated kynurenine levels and IDO1 upregulation promote an immunosuppressive microenvironment and thus allow tumor escape from immune destruction. This includes suppression of antitumorigenic CD8^+^ T cells through upregulation of the immune checkpoint PD-1/PD-L1, generation of protumorigenic regulatory T cells (Tregs), and promotion of tumor-associated M2 macrophage polarization (39, 40). Indeed, inhibition of tryptophan metabolism via IDO1 inhibitors has been FDA approved for multiple cancers either as mono- or combination therapy with anti–PD-1 (38). Interestingly, recent data by others and us suggest that both innate and adaptive immune cells, such as M2-like macrophages and CD8^+^ T cells, are also important modulators of kidney cyst growth in PKD murine models (41–43). However, the functional impact of the tryptophan pathway on cyst growth and its potential as a therapeutic target in ADPKD have not been clearly established.

Here, we utilize an orthologous ADPKD model to study tryptophan metabolism in PKD (41, 44, 45). We found both tryptophan metabolites and IDO1 expression to be elevated in ADPKD mice correlative with disease progression. Genetic loss and pharmacological inhibition of IDO1 slowed cyst growth in our model. This was associated with reduced numbers of kidney M2-like resident macrophages and Tregs, reduced expression of PD-1/PD-L1, and an increase in CD8^+^ T cell numbers within the adaptive immune cell population. Together, our data implicate tryptophan metabolism as a modifier of ADPKD progression and suggest that FDA-approved IDO1 inhibitors may present a new treatment approach for ADPKD.

## Results

### Tryptophan metabolism via IDO1 is dysregulated in Pkd1^RC/RC^ mice.

To determine whether tryptophan metabolism is abnormally regulated in murine PKD, we performed metabolomic analyses of metabolites within the tryptophan pathway using the orthologous ADPKD model C57BL/6J *Pkd1* p.R3277C (*Pkd1*^RC/RC^) (41, 44, 45). Comparing kidney metabolites of strain-, age-, and sex-matched wild type (WT) mice, we found that tryptophan levels remained stable throughout PKD progression (3 to 9 months) and comparable to WT levels (Table 1, Supplemental Figure 1, and Supplemental Table 1). However, the immunosuppressive metabolites kynurenine/kynurenic acid were significantly upregulated in *Pkd1*^RC/RC^ kidneys correlative with disease severity and independent of sex (Table 1 and Supplemental Table 1) (46). Similarly, levels of xanthurenic acid, a kynurenic acid metabolite, were elevated compared with WT. Consistent with increased production of kynurenic and xanthurenic acid, *Pkd1*^RC/RC^ kidneys displayed significantly decreased levels of picolinic acid compared with WT (Table 1 and Supplemental Table 1). Picolinic acid is an isomer of nicotinic acid, a derivative of nicotinamide, which was also significantly decreased and has been shown to slow cyst growth and improve kidney function in 2 PKD models (47). No changes were observed in levels of anthranilic acid and quinolinic acid (data not shown). Overall, these data highlight that tryptophan metabolism is dysregulated in *Pkd1*^RC/RC^ kidneys, with a shift toward the production of known immunosuppressive metabolites.

**Figure 1:**
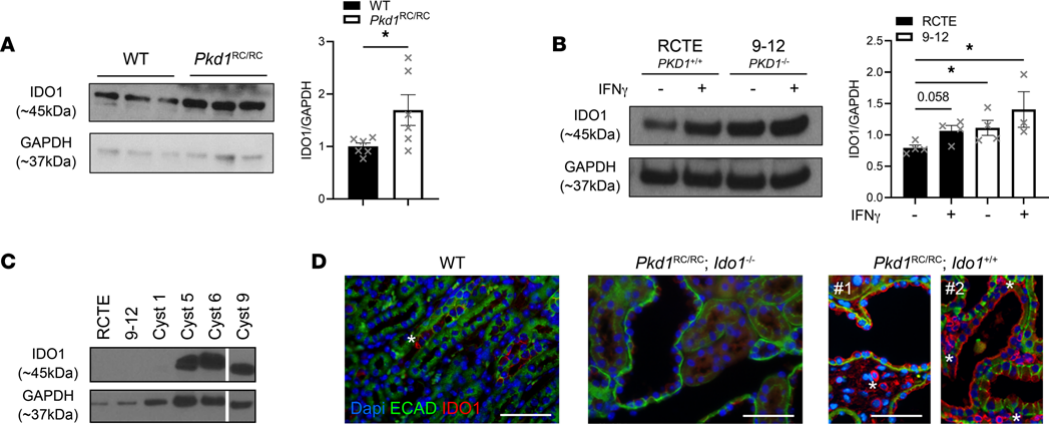
ADPKD samples present with overexpression of IDO1. (**A**) Western blot probing for IDO1 (left) and quantification (right) using WT and *Pkd1*^RC/RC^ kidney homogenates, highlighting upregulation of IDO1 in *Pkd1*^RC/RC^ kidneys compared with WT (*n* = 3 males/3 females, 9 months old). (**B**) Western blot probing for IDO1 (left) and quantification (right) of cell lysates obtained from normal renal cortical epithelial cells (RCTE, WT for *PKD1*) or 9-12 cells (null for *PKD1*) with and without IFN-γ stimulation, confirming overexpression of IDO1 in PKD-relevant human cell lines compared with control. IDO1 expression levels were further increased by the cytokine IFN-γ, which is known to be upregulated in PKD kidneys (*n* = 4 per condition). Data are presented as mean ± SEM. *P < 0.05 by unpaired *t* test (**A**) or 2-way ANOVA (Kruskal-Wallis *P* = 0.0373) with FDR Benjamini and Hochberg multiple-comparison test (**B**). Comparisons with nonsignificant statistics are not shown. (**C**) Western blot probing for IDO1 levels in epithelial cells obtained from individual cysts of ADPKD patients (each cyst was derived from a different patient; PKD genotype unknown). Most tested cysts showed high levels of IDO1 relative to IDO1 levels in RCTE or 9-12 cells (exposure time to detect IDO1 in RCTE or 9-12 cells was insufficient but IDO1 is expressed in these cell lines — see panel **B**). This provides direct clinical relevance for dysregulation of the tryptophan pathway in ADPKD patient kidneys. (**D**) Immunofluorescence images probing for IDO1 (red), E-cadherin (ECAD, green, epithelial cells), and DAPI (blue, nuclei). IDO1 is sparsely expressed in WT kidneys but upregulated in kidney cystic epithelial cells and interstitial cells of *Pkd1*^RC/RC^ kidneys. *Ido1*-knockout animals served as negative control. *Indicates IDO1-positive interstitial cells in WT or *Pkd1*^RC/RC^;*Ido1^+/+^* kidneys; #1 and #2 indicate 2 different *Pkd1*^RC/RC^;*Ido1*^+/+^ animals. Scale bars: 50 μm.

Western blot analysis of whole kidney homogenates showed increased expression of IDO1 in kidneys of *Pkd1*^RC/RC^ mice compared with WT (Figure 1A). Similar increases were observed in human 9-12 cells (*PKD1^–/–^*) compared with human renal cortical epithelial (RCTE) cells (*PKD1^+/+^*) (Figure 1B). The expression levels of IDO1 could further be amplified by stimulating RCTE or 9-12 cells with interferon gamma (IFN-γ) (Figure 1B). IFN-γ is recognized as a highly potent inducer of IDO1 via signal transducer and activator of transcription 1 (STAT-1) and/or nuclear factor kappa-light-chain-enhancer of activated B cells (NF-κB) signaling (48, 49). Importantly, we recently reported IFN-γ to be elevated in *Pkd1*^RC/RC^ kidneys and multiple murine studies accentuate aberrant activation of most STAT proteins in PKD kidneys (41, 50). Further, we found IDO1 to be expressed in primary epithelial cells derived from individual kidney cysts of ADPKD patients (Figure 1C). We confirmed elevated levels of IDO1 by immunofluorescence (Figure 1D). In WT mice, IDO1 expression was sparsely detected within interstitial cells and not in the kidney epithelium. However, *Pkd1*^RC/RC^ mice showed increased levels of IDO1 expression in cyst-lining cells and interstitial cells. Based on the literature, interstitial cells expressing IDO1 are likely macrophages or dendritic cells (DCs), which have been shown to upregulate IDO1 in various types of cancer, resulting in increased tumorigenesis (36, 49). These data suggest that the observed dysregulation of tryptophan catabolism may be attributed to overexpression of IDO1 within the cystic epithelia as well as in kidney immune cells.

### Genetic loss of Ido1 alleviates PKD severity and corrects tryptophan metabolism abnormalities.

**Figure 2:**
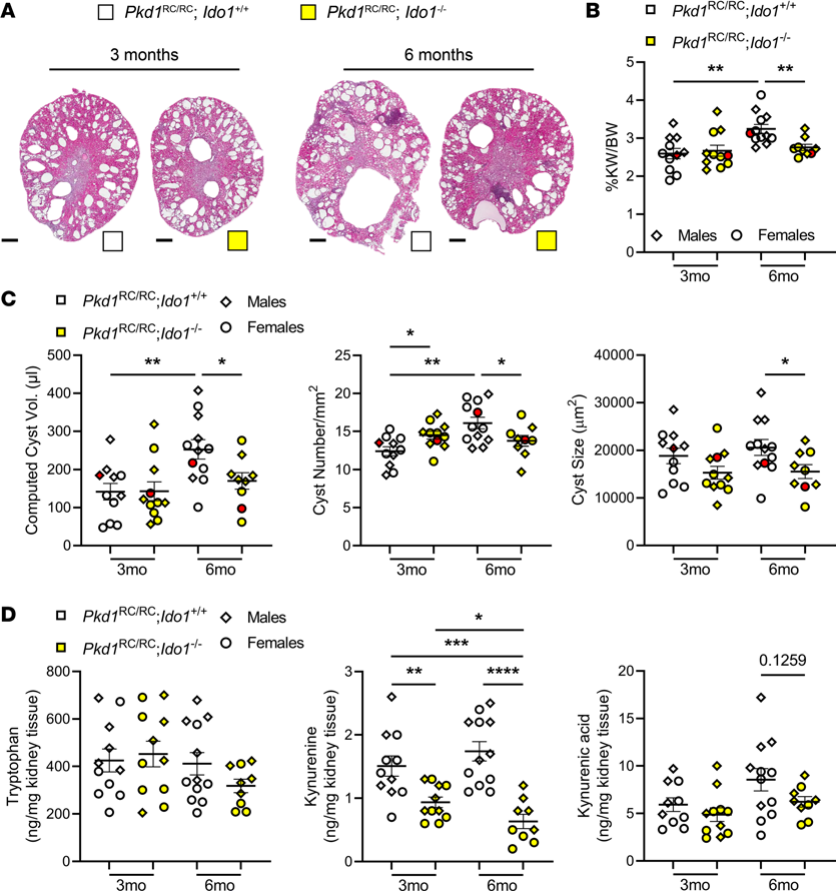
Genetic loss of *Ido1* slows cyst growth and reduces tryptophan catabolite levels in kidneys of an orthologous ADPKD model. (**A**) Kidney H&E cross sections of 3- and 6-month-old *Pkd1*^RC/RC^;*Ido1^+/+^* (white) and *Pkd1*^RC/RC^;*Ido1^–/–^* (yellow) kidneys showing overall decreased cystic disease severity in 6-month-old PKD *Ido1*-null versus *Ido1-*WT animals. Scale bars: 500 μm. Quantification of (**B**) %KW/BW and (**C**) computed cyst volume (cystic index [Supplemental Figure 2A] multiplied by kidney weight), cyst number, and cyst size in *Pkd1*^RC/RC^;*Ido1^+/+^* (white) and *Pkd1*^RC/RC^;*Ido1^–/–^* (yellow) animals, together providing statistical significance for reduced PKD severity in 6-month-old PKD *Ido1*-null versus *Ido1-*WT animals. Red data points depict the animal shown in **A**. (**D**) Quantification of significantly altered tryptophan catabolites assayed via mass spectrometry in *Pkd1*^RC/RC^;*Ido1^+/+^* (white) and *Pkd1*^RC/RC^;*Ido1^–/–^* (yellow) kidneys. Loss of *Ido1* partially corrected the observed increased levels of the immunosuppressive tryptophan catabolites, kynurenine and kynurenic acid, seen in *Pkd1*^RC/RC^ kidneys (Supplemental Figure 2D). *n* = 5 males (diamonds) and 4–7 females (circles) per genotype and time point. Data are presented as mean ± SEM. *P < 0.05, **P < 0.01, ***P < 0.001, ****P < 0.0001 by 2-way ANOVA with Tukey’s multiple-comparison test.

To functionally establish a role for tryptophan metabolism and IDO1 in PKD pathogenesis, we crossed C57BL/6J *Pkd1*^RC/RC^ mice with C57BL/6J *Ido1^–/–^* mice. When the resulting second filial generation animals (*Pkd1*^RC/RC^;*Ido1^+/+^* or *Pkd1*^RC/RC^;*Ido1^–/–^*) reached 3 or 6 months of age we evaluated histopathological/physiological features commonly analyzed in murine PKD studies (Figure 2, Supplemental Figure 2, and Supplemental Table 2). At 3 months of age we observed no difference in gross histological appearance, percentage kidney weight normalized to body weight (%KW/BW), or cystic volume/index between *Pkd1*^RC/RC^;*Ido1^+/+^* and *Pkd1*^RC/RC^;*Ido1^–/–^* mice (Figure 2, A–C, and Supplemental Figure 2A). However, at 6 months of age *Pkd1*^RC/RC^;*Ido1^–/–^* mice presented with decreased cystic kidney disease compared with *Pkd1*^RC/RC^;*Ido1^+/+^* mice, independent of sex (Figure 2, A–C, Supplemental Figure 2A, and Supplemental Table 2). Indeed, it appears that cyst growth from 3 to 6 months of age in *Pkd1*^RC/RC^;*Ido1^–/–^* mice was halted compared with controls, as evidenced by a significant reduction in kidney cyst count and size (Figure 2C). We did not detect a difference in fibrotic burden or kidney function (blood urea nitrogen [BUN]) levels [Supplemental Figure 2, B and C, and Supplemental Table 2]).

Consistent with *Ido1* loss, we observed significantly decreased levels of the immunosuppressive metabolite kynurenine in *Pkd1*^RC/RC^;*Ido1^–/–^* animals compared with controls and independent of sex (Figure 2D and Supplemental Table 2). Indeed, the kynurenine levels of 6-month-old *Pkd1*^RC/RC^;*Ido1^–/–^* mice were comparable to WT levels (Supplemental Figure 2D). Neither tryptophan nor kynurenic acid were significantly altered between the 2 *Ido1* genotypes in the setting of PKD, but we observed a possible trend toward decreased kynurenic acid levels at 6 months of age in *Pkd1*^RC/RC^;*Ido1^–/–^* animals versus controls, which was more pronounced in males versus females (Figure 2D and Supplemental Table 2).

### Ido1 loss is associated with a CME favorable to halting cyst progression.

**Figure 3:**
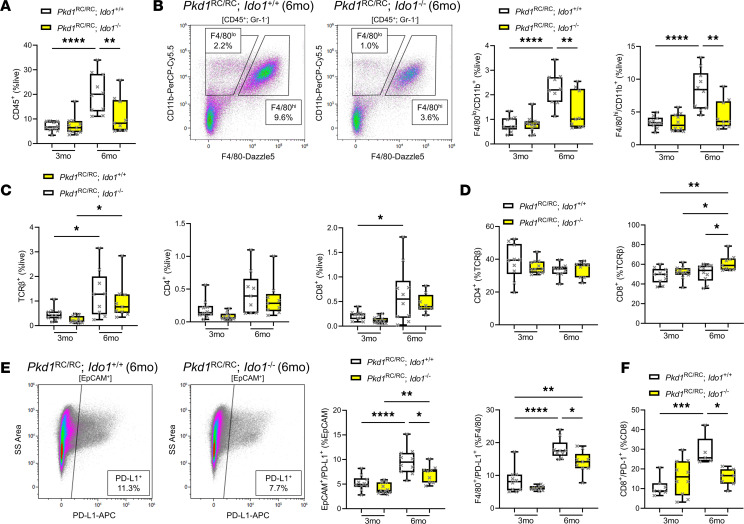
The kidney immune landscape of *Pkd1*^RC/RC^;*Ido1^–/–^* mice favors slowed PKD progression. (**A**) Flow cytometry quantification of CD45^+^ immune cells within single-cell suspensions of *Pkd1*^RC/RC^;*Ido1^+/+^* (white boxplots) and *Pkd1*^RC/RC^;*Ido1^–/–^* (yellow boxplots) kidneys showing an increase in CD45^+^ cells with PKD progression (3 to 6 months of age, *Pkd1*^RC/RC^;*Ido1^+/+^* mice) and a decrease in PKD *Ido1-*null versus WT animals at 6 months of age when reduced PKD severity was observed. (**B**) Representative flow cytometry plots indicating the gating strategy of infiltrating (F4/80^lo^, CD11b^+^) versus resident (F4/80^hi^, CD11b^+^) macrophages (left), and quantification (right), highlighting a significant increase in macrophages as disease progresses from 3 to 6 months of age in *Pkd1*^RC/RC^;*Ido1^+/+^* mice and a decrease at 6 months of age in *Pkd1*^RC/RC^;*Ido1^–/–^* mice versus WT. (**C**) Flow cytometry data quantification of all T cells (TCRβ^+^) and CD4^+^ or CD8^+^ subpopulations. The numbers of CD4^+^ or CD8^+^ cells in kidneys did not change significantly upon *Ido1* loss. (**D**) Quantification of CD4^+^ and CD8^+^ T cell numbers as percentage TCRβ^+^ cells, showing a shift in distribution of T cell subpopulations with an increase in CD8^+^ T cell numbers upon *Ido1* loss and reduced PKD severity (6 months of age). (**E**) Representative flow cytometry plot (left) and quantification (right) of immune checkpoint ligand PD-L1 expression on kidney epithelial cells (plot and quantification, EpCAM^+^) and macrophages (quantification only, F4/80^+^) indicating reduced expression in 6-month-old *Pkd1*^RC/RC^;*Ido1^–/–^* animals versus control (*Pkd1*^RC/RC^;*Ido1^+/+^*). (**F**) Quantification of immune checkpoint receptor PD-1 expression on CD8^+^ T cells showing a decrease in expression in 6-month-old *Pkd1*^RC/RC^;*Ido1^–/–^* animals versus control (*Pkd1*^RC/RC^;*Ido1^+/+^*). Whiskers show the 10th–90th percentile. *P < 0.05, **P < 0.01, ***P < 0.001, ****P < 0.0001 by 2-way ANOVA with Tukey’s multiple-comparison test. Comparisons with nonsignificant statistics are not shown. *n* = 5 males and 4–7 females per genotype/time point.

To evaluate the effect of *Ido1* loss on the cystic immune microenvironment (CME), we compared kidney immune cell types of *Pkd1*^RC/RC^;*Ido1^+/+^* and *Pkd1*^RC/RC^;*Ido1^–/–^* mice at 3 and 6 months of age using flow cytometry of kidney single-cell suspensions (Figure 3 and Supplemental Figure 3) (41, 42, 51). Overall, we found that the observed decreased PKD severity in *Pkd1*^RC/RC^;*Ido1^–/–^* mice compared with *Pkd1*^RC/RC^;*Ido1^+/+^* mice at 6 months of age was associated with lower numbers of kidney immune cells (CD45^+^; Figure 3A) and a significant decrease in infiltrating (F4/80^lo^, CD11b^+^) and resident (F4/80^hi^, CD11b^+^) macrophages, neutrophils (GR1^+^), DCs (CD11b^+^, CD11c^+^), and natural killer (NK) cells (NKp46^+^) compared with control (Figure 3B and Supplemental Figure 3, A–C). While less is known about the role of neutrophils, DCs, or NK cells in PKD, ample evidence supports a role of M2-like macrophages driving kidney/liver cyst growth in murine PKD models (42, 43, 52–58). With respect to adaptive immune cells, we did not find a change in overall T cell (TCRβ^+^, CD4^+^, or CD8^+^) numbers associated with the less severe disease observed in *Pkd1*^RC/RC^;*Ido1^–/–^* mice compared with *Pkd1*^RC/RC^;*Ido1^+/+^* mice at 6 months of age (Figure 3C). However, the distribution of T cell subtypes changed, with 6-month-old *Pkd1*^RC/RC^;*Ido1^–/–^* mice having significantly more CD8^+^ T cells compared with control (Figure 3D). Given our previously published findings that CD8^+^ T cells play an anticystogenic role in the *Pkd1*^RC/RC^ model, this increase in CD8^+^ T cell numbers supports a reduction in cyst severity in PKD *Ido1^–/–^* animals compared with control (41). This increase in CD8^+^ T cells is counterbalanced by a decrease in double-negative T cells, whose functional role has not been studied in PKD, but we and others have reported that their numbers increase in ADPKD compared with control in *Pkd1*^RC/RC^ kidneys as well as ADPKD patient kidneys (Supplemental Figure 3D) (51, 59).

Kynurenines have been reported in the cancer literature to support immune escape of tumors via engagement of the PD-1/PD-L1 immune checkpoint and differentiation of CD4^+^ T cells into immunosuppressive Tregs (60, 61). In PKD, we found activation of the PD-1/PD-L1 checkpoint as well as increased numbers of Tregs (CD4^+^, FoxP3^+^) in *Pkd1*^RC/RC^ kidneys correlative to disease progression (Supplemental Figure 4 and data not shown) (62). We observed a downward trend in kidney Treg numbers in *Pkd1*^RC/RC^;*Ido1^–/–^* animals compared with control and a significant decrease in PD-L1 expression on the kidney epithelium (EpCAM^+^, PD-L1^+^), macrophages (F4/80^+^, PD-L1^+^), and PD-1 on CD8^+^ T cells at 6 months of age (Figure 3, E and F, and Supplemental Figure 3E).

### Pharmaceutical inhibition of IDO1 slows cyst growth in a slowly and rapidly progressive model of PKD as well as changes the CME toward an anticystogenic composition.

**Figure 4:**
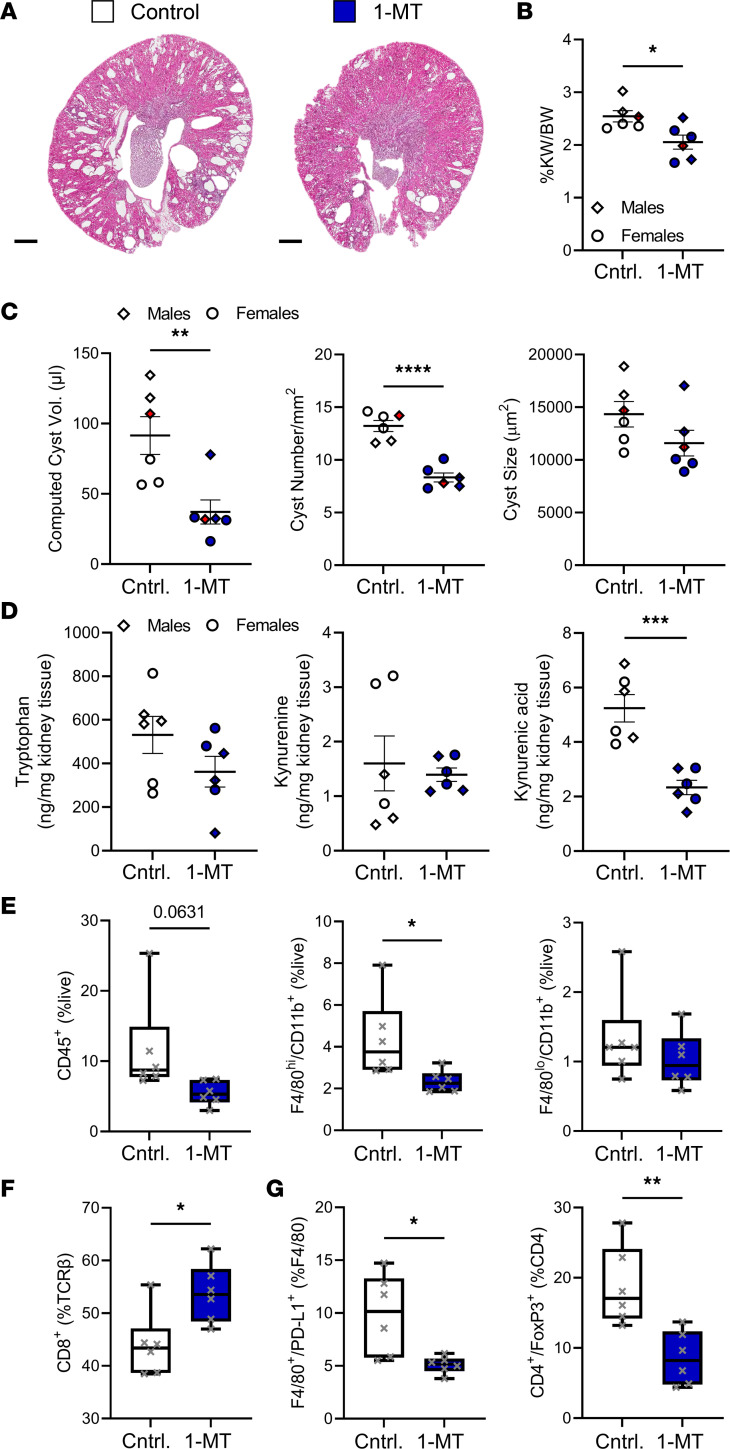
Treatment with a tryptophan analog shows therapeutic efficacy for halting slowly progressive ADPKD and is associated with changes in the immune microenvironment. Results obtained from *Pkd1*^RC/RC^ mice treated with (blue) or without (white) 1-MT. (**A**) H&E cross sections. Quantification of (**B**) %KW/BW and (**C**) cystic volume (cystic index multiplied by KW), cyst number, and cyst size (cystic index, fibrotic volume/index, and BUN can be found in Supplemental Figure 5). *Pkd1*^RC/RC^ mice treated with 1-MT show significantly reduced PKD severity compared with control (Cntrl.). (**D**) Quantification of significantly altered tryptophan catabolites assayed via mass spectrometry. 1-MT treatment significantly reduced levels of the immunosuppressive metabolite kynurenic acid. (**E** and **F**) Quantification of flow cytometry data of the 1-MT intervention experiment. 1-MT–treated animals have (**E**) reduced numbers in overall immune cells (CD45^+^), and resident macrophages (F4/80^hi^, CD11b^+^), but not infiltrating macrophages (F4/80^lo^, CD11b^+^), and (**F**) increased numbers of CD8^+^ T cells as percentage of all T cells (TCRβ^+^). (**G**) Expression of PD-L1 on macrophages (F4/80^+^) and numbers of Tregs (CD4^+^, FoxP3^+^) are reduced, both suggesting a less immunosuppressed cystic microenvironment. Scale bars: 500 μm. Treatment (1-MT): 4–7 weeks of age, *n* = 3 males (diamonds) and 3 females (circles)/group. Graphs in **B–D** show the mean ± SEM and the whiskers in the box-and-whisker plots in **E–G** box plot show 10th–90th percentiles. *P < 0.05, **P < 0.01, ***P < 0.001, ***P < 0.0001 by unpaired *t* test.

We treated 1-month-old C57BL/6J *Pkd1*^RC/RC^ mice with 400 mg/kg 1-methyl-D-tryptophan (1-MT) via oral gavage twice daily for 3 weeks (63, 64). 1-MT is a synthetic tryptophan analog/IDO pathway inhibitor (65–67). *Pkd1*^RC/RC^ mice treated with 1-MT versus control displayed significantly less severe PKD as apparent by histology, %KW/BW, cyst volume/index, and cyst number (Figure 4, A–C, Supplemental Figure 5A, and Supplemental Table 3). This treatment effect occurred independent of sex (Supplemental Table 3). Treated mice also showed a significant reduction in kidney kynurenic acid levels versus controls (Figure 4D and Supplemental Table 3). Kynurenine levels, however, remained unchanged, which may be explained by the dynamic catabolic balance between kynurenine and kynurenic acid. Treatment did not impact cyst size, fibrotic burden, or kidney function (Figure 4C, Supplemental Figure 5A, and Supplemental Table 3).

**Figure 5:**
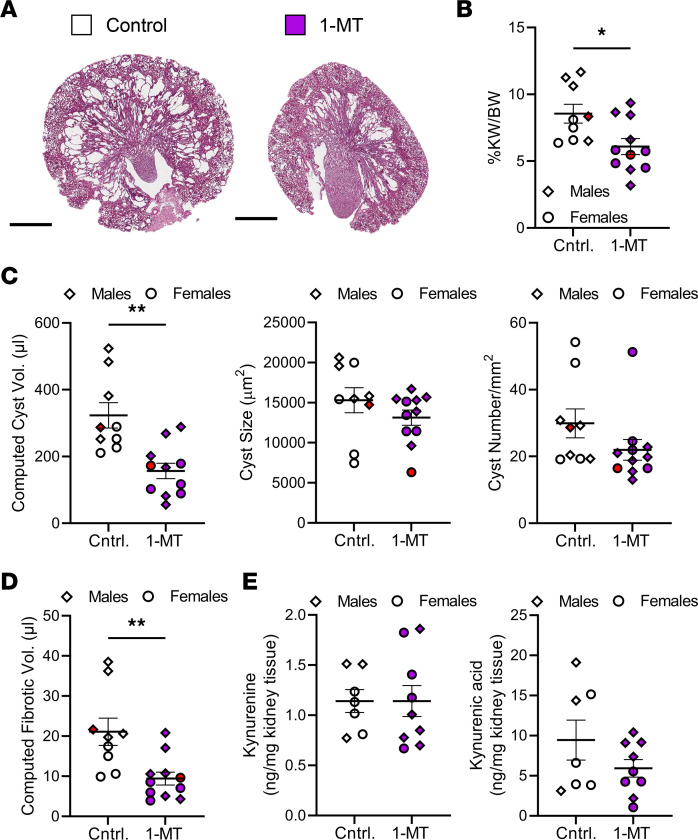
1-MT treatment slows PKD progression in a rapidly progressive, inducible PKD2 model. Results obtained from *Pax8*^rtTA^;TetO-cre;*Pkd2^fl/fl^* mice treated with (purple) or without (white) 1-MT. (**A**) H&E cross sections. Quantification of (**B**) %KW/BW, (**C**) cystic volume (cystic index multiplied by KW), cyst size, and cyst number, and (**D**) fibrotic volume (cystic index, fibrotic index, and BUN can be found in Supplemental Figure 6). *Pax8*^rtTA^;TetO-cre;*Pkd2^fl/fl^* mice treated with 1-MT show significantly reduced PKD severity compared with control (Cntrl.). (**E**) Quantification of significantly altered tryptophan catabolites assayed via mass spectrometry. 1-MT treatment results in a trend toward reduced levels of the immunosuppressive metabolite kynurenic acid. Scale bars: 1 mm. Treatment (1-MT): P12–P21, *n* = 5–6 males (diamonds) and 4–5 females (circles). Control: *n* = 3–5 males (diamonds) and 4 females (circles). Data are presented as mean ± SEM. *P < 0.05, **P < 0.01 by unpaired *t* test. Comparisons with nonsignificant statistics are not shown.

We confirmed the therapeutic efficacy of 1-MT using the juvenile induced C57BL/6J *Pax8*^rtTA^;TetO-cre;*Pkd2^fl/fl^* model (68–70). In this model, kidney-specific loss of *Pkd2* was induced via intraperitoneal (i.p.) injection of doxycycline at postnatal day 10 (P10) and P11. The mice presented with rapidly progressive PKD, with a 50% survival rate of 38.5 days (Supplemental Figure 6, A–D). Generally, females have less rapidly progressive kidney disease than males (Supplemental Figure 6, A and B). As for *Pkd1*^RC/RC^ mice, tryptophan metabolism was dysregulated in this model, as highlighted by significantly increased kynurenic acid levels in the kidney correlative with disease severity, although no difference in kynurenine was noted (Supplemental Figure 6E). *Pax8*^rtTA^;TetO-cre;*Pkd2^fl/fl^* mice were treated with 100 mg/kg 1-MT from P12 to P21 via daily i.p. injections (71–73). *Pax8*^rtTA^;TetO-cre;*Pkd2^fl/fl^* mice treated with 1-MT presented with a significant reduction in %KW/BW as well as cyst and fibrotic volume/index compared with control. Cyst size, number, and BUN trended toward a therapeutic effect of reduced PKD severity upon 1-MT treatment (Figure 5, A–D, and Supplemental Figure 6F). Treatment efficacy was noted independent of sex, although males showed a better response to 1-MT treatment compared with females (Supplemental Table 4). 1-MT treatment resulted in a trend toward reduced kidney kynurenic acid levels but did not change tryptophan or kynurenine levels (Figure 5E, Supplemental Figure 6G, and Supplemental Table 4).

As for the genetic *Ido1*-knockout model, we evaluated the impact of 1-MT treatment on the kidney immune microenvironment in *Pkd1*^RC/RC^ mice. Immune cell (CD45^+^), NK cell, DC, neutrophil, and macrophage numbers decreased in kidneys of 1-MT–treated animals versus controls, although not all decreases reached significance (Figure 4E and Supplemental Figure 5, B–D). Interestingly, upon 1-MT treatment, the number of kidney-resident macrophages (F4/80^hi^, CD11b^+^) preferentially decreased, while the number of kidney-infiltrating macrophages (F4/80^lo^, CD11b^+^) remained unchanged (Figure 4E). 1-MT treatment, similarly to genetic loss of *Ido1*, resulted in a shift of the T cell (TCRβ^+^) population toward CD8^+^ T cells and a decrease in double-negative T cell numbers within the kidney; no changes were detected in overall T cell numbers or subtypes (Figure 4F and Supplemental Figure 5F). 1-MT–treated animals also had significantly reduced expression of PD-L1 on macrophages (F4/80^+^), and a trend toward reduced expression on epithelial cells (EpCAM^+^) (Figure 4G and Supplemental Figure 5E). We also observed a significant decrease in Treg (CD4^+^, FoxP3^+^) numbers within the kidneys of 1-MT–treated *Pkd1*^RC/RC^ mice compared with control, which reinforces the idea of kynurenines driving Treg differentiation (Figure 4G). Together, these data provide preclinical support for use of IDO1 inhibitors as a PKD therapeutic and highlight that pharmaceutical inhibition of IDO1 mimics the immunomodulatory phenotypes observed in the genetic model.

## Discussion

Tryptophan and its metabolites regulate a variety of physiological processes, including cell growth/maintenance and neuronal function. However, greater than 95% of tryptophan is a substrate for the kynurenine pathway, which controls hyperinflammation and long-term immune tolerance (74, 75). While IDO1 plays a minor role in metabolizing tryptophan to kynurenine under normal conditions, IDO1 levels and IDO1-dependent tryptophan metabolism in macrophages, DCs, and epithelial cells is potently induced by inflammatory signals. These include IFN-γ, IL-6, and TNF-α, all of which have been found by others and us to be elevated in murine models of PKD or ADPKD patient cyst fluid (41, 75–77). Indeed, our in vitro studies confirm that treatment with recombinant IFN-γ induces IDO1 expression in cystic epithelial cells. Consistently, we observed increased levels of kynurenines and IDO1 correlative to disease severity and increased IDO1 staining in interstitial cells as well as the cystic epithelium of *Pkd1*^RC/RC^ mice. These results parallel our recent findings that kynurenine and kynurenic acid are significantly accumulated in plasma collected from children and adults with ADPKD compared with healthy individuals (78).

In cancer, kynurenine/kynurenic acid and IDO1 levels regulate immunosuppression through suppression of antitumorigenic immune cells (DCs, NK, and effector T cells), expansion of protumorigenic immune cells (Tregs, M2 macrophages, and myeloid-derived suppressor cells), and upregulation of immune checkpoints (PD-1/PD-L1, CTLA4/CD80 or CD86) (61, 79, 80). To date, the functional role of DCs, NK cells, and the different CD4^+^ T cell subtypes in PKD progression have not been well defined. However, urinary CD4^+^ T cell numbers have been shown to correlate with eGFR decline in ADPKD patients, and our recent publication suggests that granzyme B– or IFN-γ–producing T cells may be critical to mediating injury-driven PKD (59, 81). Similarly, kidney M2-like macrophages are known to drive PKD, and we have shown that loss of CD8^+^ T cells enhances cyst growth in the *Pkd1*^RC/RC^ model (41, 52–56, 82–84). Here, we further provide data showing that kidney Treg numbers are increased in *Pkd1*^RC/RC^ mice compared with WT, suggesting they may play a role in PKD. Since genetic loss of *Ido1* or 1-MT treatment in our PKD model not only reduced kidney kynurenine/kynurenic acid levels, but also slowed cyst growth, we hypothesized inhibition of immunosuppressive pathways to be an underlying mechanism. In line with this hypothesis, we observed decreased numbers of kidney macrophages and increased numbers of CD8^+^ T cells within the adaptive immune cell population (TCRβ^+^) in the kidneys of *Pkd1*^RC/RC^ *Ido1*-knockout or 1-MT–treated animals compared with controls. Further, we observed downregulation of PD-1/PD-L1 expression and a reduction in Treg numbers, all favorable for a CME that supports slowed cyst growth. We also observed fewer DCs, NK cells, and neutrophils in *Pkd1*^RC/RC^ mice when tryptophan metabolism was inhibited, but the functional impact of these microenvironmental changes is less clear. A key limitation of our CME analyses is that we only investigated immune cell numbers and not their function. It is also unclear whether our observed changes in CME composition are directly due to IDO1 inhibition or a consequence of reduced PKD severity. Future mechanistic studies are needed to disjoin these 2 observations and decipher cell types critical to IDO1-mediated PKD pathogenesis. Conditional, cell-type-specific *Ido1* loss in the setting of PKD would establish which cells are key producers of kynurenine/kynurenic acid as well as outline whether the pathogenic impact of IDO1 overexpression and/or high kynurenine/kynurenic acid levels is predominantly mediated by immunosuppression or cell-autonomous effects within the cystic kidney epithelium. To that extent, kynurenines may drive proproliferative pathways within the kidney epithelium through binding to the aryl hydrocarbon receptor (AhR), as has been described in cancer (85). AhR interacts with a multitude of proteins found to be key modulators of PKD, including mechanistic target of rapamycin, mitogen-activated protein kinases, sirtuin-1, and NF-κB (86–90). Interestingly, we found that treatment with 1-MT decreased the proliferative rate of 9-12 cells to a greater extent than that of RCTE cells, suggesting that the therapeutic effect of IDO1 inhibition may go beyond immunomodulation (Supplemental Figure 7).

There are several other limitations in this study. Firstly, the data suggest kidney accumulation of both kynurenine and kynurenic acid driving PKD pathology and immunosuppression. Future studies will need to test whether individual administration of either of these tryptophan metabolites to murine PKD models is sufficient to modulate kidney cyst growth and/or immune cell function. In addition, other tryptophan metabolites might be critical to the pathology. For example, both 3-hydroxyanthranilic acid and quinolinic acid have been shown to lead to effector T cell apoptosis, hence contributing to an immunosuppressive milieu (91). Neither of these metabolites, however, were significantly altered in our model of ADPKD. Secondly, it is unclear whether differential IDO1 levels are the sole driver of kynurenine/kynurenic acid accumulation in ADPKD. Indeed, analysis of key tryptophan-metabolizing enzymes in kidneys of *Pkd1*^RC/RC^ versus WT mice revealed significantly elevated levels of IDO2 and kynurenine amino transferase (KAT) as well as decreased levels of kynurenine 3-monooxygenase (KMO) (*P* = 0.08) and kynureninase (KYNU) (*P* = 0.01), all potentially contributing to kynurenine/kynurenic acid accumulation in the kidney (Supplemental Figures 1 and 8). We also did not assay IDO1 enzymatic activity, which could be enhanced in the setting of ADPKD. A detailed dissection of the different tryptophan catabolism pathway junctions and evaluation of enzyme activities will be required to better delineate which aspect of dysregulated tryptophan metabolism is critical to driving kidney cyst growth. These studies are essential to better understand how to ideally target the pathway for clinical translation. Finally, a better understanding of what drives increased IDO1 expression in PKD would facilitate therapeutic targeting of the pathway. While we show that IFN-γ may contribute to increased IDO1 levels, there are multiple other pathways of IDO1 induction that were not assayed. For example, cyclooxygenase-2 (COX2) and prostaglandin E2 (PGE2) activate IDO transcription via the phosphatidylinositol-3-kinase pathway (49). Interestingly, levels of both COX2 and PGE2 are increased in PKD models and patient cyst fluid, and inhibition of COX2 has been shown to ameliorate PKD in *Anks6^+/–^* (Han:SPRD Cy/+) rats, a nonorthologous ADPKD model, and in *Pkd2*^WS25/−^ mice. However, the selective COX2 inhibitor celecoxib did not impact disease severity in *Pkd1*^RC/RC^ mice (92–94). In addition, in a series of tumor cell lines, IDO1 expression was shown to be driven by an autocrine positive feedback loop via the activation of AhR by kynurenine (49). We did not evaluate AhR-dependent transcripts within this study.

It remains unclear why in our genetic model of IDO1 loss we did not observe a phenotypic difference in PKD severity until 6 months of age compared with control, or why some of the observed changes in the CME differed between genetic loss or pharmacological inhibition of IDO1. Regarding the latter, it is interesting that only pharmacological inhibition of IDO1 reduced the number of kidney Tregs, which we hypothesize to be drivers of cyst growth. Similarly, pharmacological inhibition of IDO1 resulted in a selective reduction in resident macrophages and not infiltrating macrophages, whereas genetic loss decreased both populations within the kidney. The data on the contribution of infiltrating macrophages to PKD progression are inconsistent, and a single study suggests that resident macrophages promote cyst growth in a nonorthologous model of PKD (56, 58, 95). The role of these populations has not been studied in the slowly progressive *Pkd1*^RC/RC^ model. Further, our data suggest that treatment with 1-MT is more effective in slowing PKD in our *Pkd1*^RC/RC^ versus genetic *Ido1* loss. This could be due to treatment with 1-MT impacting additional pathways. For example, 1-MT has been shown to have some affinity for IDO2, which is also increased in levels within the ADPKD kidney (Supplemental Figure 8) (96). Further, 1-MT has been shown to directly modulate AhR activity as well as mTORC1/autophagy and FoxP3 expression in T cells, all of which are relevant to PKD (97).

Interestingly, like annual changes in kidney growth and eGFR decline in ADPKD patients, kynurenine levels have also been positively associated with body mass index (10, 98, 99). Since tryptophan is an essential amino acid, the impact of various dietary regimens on kynurenine levels has been studied in animal models. Caloric restriction and a ketogenic diet resulted in downregulation of kynurenines, both of which have also been found to slow PKD progression in murine models (16–18, 100, 101).

In conclusion, our data provide convincing evidence that the kynurenine pathway and IDO1 are dysregulated in PKD and that targeting the pathway provides a therapeutic platform for disease treatment. 1-MT (NLG-8189) as well as other IDO1 inhibitors such as epacadostat, which has higher affinity for IDO1 than 1-MT, are being tested in multiple clinical trials for cancer. These inhibitors have been found to be well tolerated with minimal toxicity (102, 103). While the antitumor efficacy of IDO1 inhibitors alone was found to be limited, studies report synergistic effects if combined with immune checkpoint inhibitors (102, 103). Our data strongly support the testing of FDA-approved IDO1 inhibitors in long-term preclinical ADPKD studies, with a goal of clinical translation. Similarly, we believe that the impact of dysregulated metabolism on immune cell function in PKD warrants further investigation and could emerge as a promising new therapeutic platform for combination treatment approaches with epithelium-centric drugs such as tolvaptan.

## Methods

Full methods are available in the supplemental material.

### Mouse models.

The homozygous C57BL/6J p.R3277C (*Pkd1*^RC/RC^) (41, 44, 45) model was crossed with C57BL/6 *Ido1^–/–^* (Jackson Laboratory, stock 005867) to generate C57BL/6 *Pkd1*^RC/RC^;*Ido1^–/–^* and C57BL/6 *Pkd1*^RC/RC^;*Ido1^+/+^* animals. The C57BL/6J *Pax8*^rtTA^;TetO-cre;*Pkd2^fl/fl^* model was received from the NIH NIDDK PKD Research Resource Consortium (68). *Pkd2* loss was induced by i.p. injection of doxycycline at 40 mg/kg on P10 and 50 mg/kg on P11.

### IDO1 inhibition.

One-month-old C57BL/6J *Pkd1*^RC/RC^ mice were treated by oral gavage for 3 weeks: twice daily with 400 mg/kg 1-MT (Sigma-Aldrich, 452483, IDO1 inhibitor) or 0.5% hydroxypropyl methyl cellulose/0.1% Tween 80 (control). P12–P21 C57BL/6J *Pax8*^rtTA^;TetO-cre;*Pkd2^fl/fl^* mice were i.p. injected daily with 100 mg/kg 1-MT or buffered PBS as control (1N HCl adjusted to pH 7.0 with sterile PBS adjusted to pH 7.0 with 1N HC1 and 1M NaOH).

### Human samples.

Deidentified ADPKD patient cyst cells were obtained from the Baltimore Polycystic Kidney Disease Research and Clinical Core Center (NIDDK, P30DK090868).

### Cell culture.

Cell lines (RCTE and 9-12) used have been previously described (104). Cyst cells and cell lines were grown in DMEM/F12 (50:50) plus L-glutamine, 15 mM HEPES (Corning), 10% fetal bovine serum (VWR International), and 1% penicillin-streptomycin (Corning).

### IFN-γ stimulation.

RCTE and 9-12 cells were treated for 24 hours with recombinant human IFN-γ (PeproTech, 300-02) at 100 ng/mL or with PBS control.

### Cell viability assay.

RCTE and 9-12 cells were seeded in 96-well plates for 3-(4,5-dimethylthiazol-2-yl)-2,5-diphenyltetrazolium (MTT; Thermo Fischer Scientific, M6494) assay. Cells were treated with vehicle (PBS) or 1-MT dissolved in PBS. Subsequently, cells were incubated in MTT solution (5 mg/mL) and purple formazan was measured per manufacturer’s instructions.

### Kidney function analyses.

BUN was measured following the manufacturer’s protocol (QuantiChrom Urea Assay Kit, BioAssay Systems, 501078333).

### Histomorphometric analysis.

Kidney cystic index, cyst size, and cyst number were analyzed using a custom-built NIS-Elements AR v4.6 macro (Nikon). Fibrotic area was analyzed from picrosirius red–stained kidney sections and visualized using an Olympus BX41 microscope with a linear polarizer. Images were obtained and quantified as previously described (41).

### Immunofluorescent labeling.

Paraffin-embedded tissues were processed and stained as previously described (41). Primary and secondary antibodies were the following: 1° anti–mouse IDO1 (clone mIDO-48, BioLegend; 1:50), 2° Alexa Fluor 594–goat anti–rabbit IgG (Life Technologies; 1:1000); 1° anti–mouse E-cadherin (clone 36, BD Transduction Laboratories; 1:100), and 2° Alexa Fluor 488–goat anti–rat IgG (Life Technologies; 1:1000). Sections were mounted with Vectashield mounting medium with DAPI (Vector Labs) and visualized with a Nikon Eclipse Ti microscope.

### Western blotting.

Whole cell lysates from cell lines, cells derived from human cysts, or homogenized mouse kidney tissue were prepared in RIPA cell lysis buffer with protease inhibitor (P8340, Sigma-Aldrich). Protein samples were resolved by SDS-PAGE and transferred to PVDF membranes. Membranes were incubated with primary antibodies overnight and detected using HRP-linked secondary antibodies and ECL detection reagents. Primary antibodies against the following proteins were used: IDO1 (clone mIDO-48, BioLegend, 122402; 1:400), IDO2 (clone 1HC, Novus Biologicals, NBP2-21641; 1:500), KYNU (clone 771312, R&D Systems, MAB7389; 1:500), KMO (clone 2493A, R&D Systems, MAB8050-SP; 1:500), KAT (clone C-7, Santa Cruz Biotechnology, sc-374531; 1:500), and GAPDH (polyclonal FL-335, Santa Cruz Biotechnology, sc-25778; 1:500). Secondary antibodies were the following: anti-rat–HRP (1:5000), anti-rabbit–HRP (1:5000), and anti-mouse–HRP (1:10,000). Densitometry on x-ray films was quantified using ImageJ (NIH).

### Single-cell suspensions.

Single cell suspensions were prepared as previously described (41). Kidneys harvested from terminal dissection were mechanically dissociated and digested in DMEM/F12, Liberase TL, and DNase I at 37°C for 30 minutes. Dissociation was completed using an 18G needle and quenched using FA3 buffer. Cells were passed through a 100 μm filter, incubated with red blood cell lysis buffer, and then passed through a 70 μm filter. Cells were suspended in FA3 buffer for flow cytometry staining.

### Flow cytometry.

Flow cytometry was performed as previously described (41). Detailed methods, antibodies used, and gating strategies are outlined in the supplemental material. The blocked, viability dye–stained cell suspension was split in 2 and used for different flow cytometry panels. Panel 1 (innate immune and epithelial cells): CD45-FITC, CD11c-PE, F4/80-PE/Dazzle 594, CD11b–PerCP-Cy5.5, Gr-1–PE/Cy7, PD-L1–APC, MHCII–DyLight 680, EpCAM–APC–eFluor 780, and NKp46–eFluor 450. Panel 2 (T cells): CD44-FITC, PD-1–PE, CD45–PE-CF594, TCRβ–PE-Cy5, CD69–PE-Cy7, CD8–Alexa Fluor 700, CD4-APC/Cy7, Ki-67–APC, and FoxP3–eFluor 450. Stained cells were analyzed on the Gallios Flow Cytometer. All data were analyzed using Kaluza Analysis v2.1 (Beckman Coulter).

### Metabolomics — liquid chromatography–tandem mass spectrometry.

Semiquantitative targeted metabolomics was performed following a validated approach (26, 105). Kidney tissue was homogenized in 80% (v/v) cooled methanol, incubated for protein precipitation, dried in a SpeedVac concentrator centrifuge (Savant, Thermo Fisher Scientific), and reconstituted in water/methanol (80:20 v/v). Selected multiple reaction monitoring of 250 metabolites using a positive/negative ion-switching high-performance liquid chromatography-tandem mass spectrometry (LC-MS/MS) (5500 QTRAP HPLC–MS/MS21) was used for analysis. MultiQuant (v2.1.1, Sciex) software was used for data processing of the 250 unique metabolites.

Kynurenines were analyzed using a modified protocol (106). Frozen tissue was homogenized in 0.5 mL formic acid (10% in water/methanol 30:70, v/v). The extraction solution was enriched with isotope-labeled internal standards and samples were vortexed and centrifuged at 26,000*g* for 20 minutes. The supernatant was transferred into HPLC vials with glass inserts. LC-MS/MS was performed on an Agilent Technologies 1200 HPLC system connected to an ABSCIEX 5500 QTRAP mass spectrometer equipped with a turbo ion spray source operated in electrospray mode. LC separation was carried out on an Atlantis T3 3 μm (2.1 × 50 mm) column (Waters Corp.) using a mobile phase consisting of 0.1% formic acid in water (solvent A) and acetonitrile (solvent B). All analytes were detected in positive ion multiple reaction monitoring mode.

Data analysis was performed using MetaboAnalyst v4.0 (107).

### Statistics.

Data were analyzed using Prism 9 (GraphPad Software). Data are depicted as mean ± SEM or box-and-whisker plots with whiskers showing the 10th-90th percentile and the box extending from the 25th to the 75th percentile. The middle line depicts the median and all data points excluding outliers as determined by ROUT testing are shown; single data points are depicted. Analyses were performed as unpaired 2-tailed *t* test or 2-way ANOVA with Tukey’s multiple-comparison test including ROUT (*Q* = 1%) outlier testing. A *P* value of less than 0.05 was considered significant: *P < 0.05, **P < 0.01, ***P < 0.001, and ****P < 0.0001.

### Study approval.

All animal procedures were performed in an AAALAC-accredited facility in accordance with the NIH *Guide for the Care and Use of Laboratory Animals* (108) and approved by the University of Colorado Anschutz Medical Campus Institutional Animal Care and Use Committee (protocol nos. 33 and 685).

## Author contributions

DTN, EKK, and KH designed the research study and BYG, MBC, and RAN provide guidance on the study design. DTN, EKK, ND, MLTM, ETC, JK, and KH conducted the experiments and acquired the data.DTN, JK, and KH analyzed the data. RAN, JK, and KH wrote the manuscript. EKK, ND, BYG, MBC, and ETC provide feedback on the manuscript. Authorship order between DTN and EKK, co–first authors, was decided based on overall time and intellectual contribution to the study.

## Supplementary Material

Supplemental data

## Figures and Tables

**Table 1:**
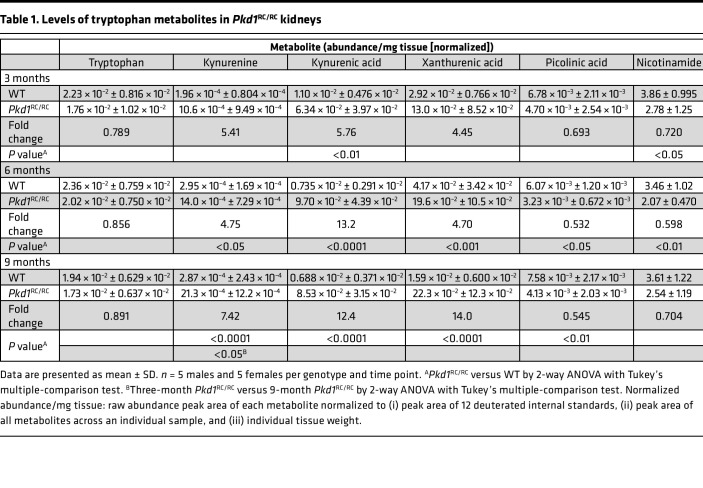
Levels of tryptophan metabolites in *Pkd1*^RC/RC^ kidneys

## Version 1 11/24/2022

In-Press Preview

## Version 2 01/10/2023

Electronic publication

## References

[1] Bergmann C (2018). Polycystic kidney disease. Nat Rev Dis Primers.

[2] Torres VE (2007). Autosomal dominant polycystic kidney disease. Lancet.

[3] Reule S (2014). ESRD from autosomal dominant polycystic kidney disease in the United States, 2001-2010. Am J Kidney Dis.

[4] https://www.niddk.nih.gov/about-niddk/strategic-plans-reports/usrds/prior-data-reports/2018.

[5] Torres VE (2012). Tolvaptan in patients with autosomal dominant polycystic kidney disease. N Engl J Med.

[6] Torres VE (2017). Tolvaptan in later-stage autosomal dominant polycystic kidney disease. N Engl J Med.

[7] Cornec-Le Gall E (2018). Genetic complexity of autosomal dominant polycystic kidney and liver diseases. J Am Soc Nephrol.

[8] Hopp K (2020). Detection and characterization of mosaicism in autosomal dominant polycystic kidney disease. Kidney Int.

[9] Harris PC (2006). Cyst number but not the rate of cystic growth is associated with the mutated gene in autosomal dominant polycystic kidney disease. J Am Soc Nephrol.

[10] Nowak KL (2018). Overweight and obesity are predictors of progression in early autosomal dominant polycystic kidney disease. J Am Soc Nephrol.

[11] Torres VE (2017). Dietary salt restriction is beneficial to the management of autosomal dominant polycystic kidney disease. Kidney Int.

[12] Takakura A (2009). Renal injury is a third hit promoting rapid development of adult polycystic kidney disease. Hum Mol Genet.

[13] Nowak KL, Hopp K (2020). Metabolic reprogramming in autosomal dominant polycystic kidney disease: evidence and therapeutic potential. Clin J Am Soc Nephrol.

[14] Menezes LF, Germino GG (2019). The pathobiology of polycystic kidney disease from a metabolic viewpoint. Nat Rev Nephrol.

[15] Padovano V (2018). Metabolism and mitochondria in polycystic kidney disease research and therapy. Nat Rev Nephrol.

[16] Warner G (2016). Food restriction ameliorates the development of polycystic kidney disease. J Am Soc Nephrol.

[17] Kipp KR (2016). A mild reduction of food intake slows disease progression in an orthologous mouse model of polycystic kidney disease. Am J Physiol Renal Physiol.

[18] Torres JA (2019). Ketosis ameliorates renal cyst growth in polycystic kidney disease. Cell Metab.

[19] Rowe I (2013). Defective glucose metabolism in polycystic kidney disease identifies a new therapeutic strategy. Nat Med.

[20] Chiaravalli M (2016). 2-Deoxy-D-glucose ameliorates PKD progression. J Am Soc Nephrol.

[21] Lakhia R (2018). PPARα agonist fenofibrate enhances fatty acid β-oxidation and attenuates polycystic kidney and liver disease in mice. Am J Physiol Renal Physiol.

[22] Flowers EM (2018). Lkb1 deficiency confers glutamine dependency in polycystic kidney disease. Nat Commun.

[23] Trott JF (2018). Arginine reprogramming in ADPKD results in arginine-dependent cystogenesis. Am J Physiol Renal Physiol.

[24] Ramalingam H (2021). A methionine-Mettl3-N^6^-methyladenosine axis promotes polycystic kidney disease. Cell Metab.

[25] Grams ME (2017). Metabolomic alterations associated with cause of CKD. Clin J Am Soc Nephrol.

[26] Baliga MM (2021). Metabolic profiling in children and young adults with autosomal dominant polycystic kidney disease. Sci Rep.

[27] Badawy AA (2017). Kynurenine pathway of tryptophan metabolism: regulatory and functional aspects. Int J Tryptophan Res.

[28] Schmiedel BJ (2018). Impact of genetic polymorphisms on human immune cell gene expression. Cell.

[29] Monaco G (2019). RNA-seq signatures normalized by mRNA abundance allow absolute deconvolution of human immune cell types. Cell Rep.

[30] Debnath S (2017). Tryptophan metabolism in patients with chronic kidney disease secondary to type 2 diabetes: relationship to inflammatory markers. Int J Tryptophan Res.

[31] Munipally PK (2011). Evaluation of indoleamine 2,3-dioxygenase expression and kynurenine pathway metabolites levels in serum samples of diabetic retinopathy patients. Arch Physiol Biochem.

[32] Prendergast GC (2011). Cancer: why tumours eat tryptophan. Nature.

[33] Rhee EP (2013). A combined epidemiologic and metabolomic approach improves CKD prediction. J Am Soc Nephrol.

[34] Huang JY (2018). A prospective evaluation of serum kynurenine metabolites and risk of pancreatic cancer. PLoS One.

[35] Routy JP (2016). The kynurenine pathway is a double-edged sword in immune-privileged sites and in cancer: implications for immunotherapy. Int J Tryptophan Res.

[36] Brochez L (2017). The rationale of indoleamine 2,3-dioxygenase inhibition for cancer therapy. Eur J Cancer.

[37] Seeger-Nukpezah T (2015). The hallmarks of cancer: relevance to the pathogenesis of polycystic kidney disease. Nat Rev Nephrol.

[38] Liu M (2018). Targeting the IDO1 pathway in cancer: from bench to bedside. J Hematol Oncol.

[39] Bader JE (2020). Targeting metabolism to improve the tumor microenvironment for cancer immunotherapy. Mol Cell.

[40] Patel CH (2019). Targeting metabolism to regulate immune responses in autoimmunity and cancer. Nat Rev Drug Discov.

[41] Kleczko EK (2018). CD8^+^ T cells modulate autosomal dominant polycystic kidney disease progression. Kidney Int.

[42] Zimmerman KA (2020). Role of chemokines, innate and adaptive immunity. Cell Signal.

[43] Li Z (2021). Resident macrophages in cystic kidney disease. Kidney360.

[44] Arroyo J (2021). The genetic background significantly impacts the severity of kidney cystic disease in the Pkd1^RC/RC^ mouse model of autosomal dominant polycystic kidney disease. Kidney Int.

[45] Hopp K (2012). Functional polycystin-1 dosage governs autosomal dominant polycystic kidney disease severity. J Clin Invest.

[46] Wirthgen E (2017). Kynurenic acid: the Janus-faced role of an immunomodulatory tryptophan metabolite and its link to pathological conditions. Front Immunol.

[47] Zhou X (2013). Sirtuin 1 inhibition delays cyst formation in autosomal-dominant polycystic kidney disease. J Clin Invest.

[48] Robinson CM (2003). Synergistic transcriptional activation of indoleamine dioxygenase by IFN-gamma and tumor necrosis factor-alpha. J Interferon Cytokine Res.

[49] Meireson A (2020). IDO expression in cancer: different compartment, different functionality?. Front Immunol.

[50] Strubl S (2020). STAT signaling in polycystic kidney disease. Cell Signal.

[51] Nemenoff RA (2019). Renal double negative T cells: unconventional cells in search of a function. Ann Transl Med.

[52] Swenson-Fields KI (2013). Macrophages promote polycystic kidney disease progression. Kidney Int.

[53] Karihaloo A (2011). Macrophages promote cyst growth in polycystic kidney disease. J Am Soc Nephrol.

[54] Locatelli L (2016). Macrophage recruitment by fibrocystin-defective biliary epithelial cells promotes portal fibrosis in congenital hepatic fibrosis. Hepatology.

[55] Viau A (2018). Cilia-localized LKB1 regulates chemokine signaling, macrophage recruitment, and tissue homeostasis in the kidney. EMBO J.

[56] Cassini MF (2018). Mcp1 promotes macrophage-dependent cyst expansion in autosomal dominant polycystic kidney disease. J Am Soc Nephrol.

[57] Zimmerman KA (2020). Interferon regulatory factor-5 in resident macrophage promotes polycystic kidney disease. Kidney360.

[58] Zimmerman KA (2019). Tissue-resident macrophages promote renal cystic disease. J Am Soc Nephrol.

[59] Zimmerman KA (2019). Urinary T cells correlate with rate of renal function loss in autosomal dominant polycystic kidney disease. Physiol Rep.

[60] Mezrich JD (2010). An interaction between kynurenine and the aryl hydrocarbon receptor can generate regulatory T cells. J Immunol.

[61] Liu Y (2018). Tumor-repopulating cells induce PD-1 expression in CD8^+^ T cells by transferring kynurenine and AhR activation. Cancer Cell.

[62] https://www.asn-online.org/education/kidneyweek/archives/KW19Abstracts.pdf.

[63] Pigott E (2014). 1-Methyl-tryptophan synergizes with methotrexate to alleviate arthritis in a mouse model of arthritis. Autoimmunity.

[64] Zeng J (2009). Prevention of spontaneous tumor development in a ret transgenic mouse model by ret peptide vaccination with indoleamine 2,3-dioxygenase inhibitor 1-methyl tryptophan. Cancer Res.

[65] Gunther J (2019). Limitations and off-target effects of tryptophan-related IDO inhibitors in cancer treatment. Front Immunol.

[66] Cady SG, Sono M (1991). 1-Methyl-DL-tryptophan, beta-(3-benzofuranyl)-DL-alanine (the oxygen analog of tryptophan), and beta-[3-benzo(b)thienyl]-DL-alanine (the sulfur analog of tryptophan) are competitive inhibitors for indoleamine 2,3-dioxygenase. Arch Biochem Biophys.

[67] Hong R (2018). Selective inhibition of IDO1, D-1-methyl-tryptophan (d-1mt), effectively increased EpCAM/CD3-bispecific BiTE antibody MT110 efficacy against IDO1^hi^ breast cancer via enhancing immune cells activity. Int Immunopharmacol.

[68] Garcia-Gonzalez MA (2010). Pkd1 and Pkd2 are required for normal placental development. PLoS One.

[69] Traykova-Brauch M (2008). An efficient and versatile system for acute and chronic modulation of renal tubular function in transgenic mice. Nat Med.

[70] Perl AK (2002). Early restriction of peripheral and proximal cell lineages during formation of the lung. Proc Natl Acad Sci U S A.

[71] Taher YA (2008). Indoleamine 2,3-dioxygenase-dependent tryptophan metabolites contribute to tolerance induction during allergen immunotherapy in a mouse model. J Allergy Clin Immunol.

[72] Chen X (2006). Indoleamine 2,3-dioxygenase (IDO) is involved in promoting the development of anterior chamber-associated immune deviation. Immunol Lett.

[73] Sakurai K (2002). Effect of indoleamine 2,3-dioxygenase on induction of experimental autoimmune encephalomyelitis. J Neuroimmunol.

[74] Platten M (2019). Tryptophan metabolism as a common therapeutic target in cancer, neurodegeneration and beyond. Nat Rev Drug Discov.

[75] Sorgdrager FJH (2019). Tryptophan metabolism in inflammaging: from biomarker to therapeutic target. Front Immunol.

[76] Munn DH (2006). Indoleamine 2,3-dioxygenase, tumor-induced tolerance and counter-regulation. Curr Opin Immunol.

[77] Zhai L (2015). Molecular pathways: targeting IDO1 and other tryptophan dioxygenases for cancer immunotherapy. Clin Cancer Res.

[78] Klawitter J Kynurenines in polycystic kidney disease. J Nephrol.

[79] Wang XF (2014). The role of indoleamine 2,3-dioxygenase (IDO) in immune tolerance: focus on macrophage polarization of THP-1 cells. Cell Immunol.

[80] Hornyak L (2018). The role of indoleamine-2,3-dioxygenase in cancer development, diagnostics, and therapy. Front Immunol.

[81] Song CJ (2022). A comprehensive immune cell atlas of cystic kidney disease reveals the involvement of adaptive immune cells in injury-mediated cyst progression in mice. J Am Soc Nephrol.

[82] Mrug M (2008). Overexpression of innate immune response genes in a model of recessive polycystic kidney disease. Kidney Int.

[83] Li Z CD206+ resident macrophages are a candidate biomarker for renal cystic disease in preclinical models and patients with ADPKD. Dis Model Mech.

[84] Zimmerman KA (2020). Interferon regulatory factor 5 in resident macrophage promotes polycystic kidney disease. Kidney360.

[85] Kwiatkowska I (2021). Not only immune escape-the confusing role of the TRP metabolic pathway in carcinogenesis. Cancers (Basel).

[86] Diry M (2006). Activation of the dioxin/aryl hydrocarbon receptor (AhR) modulates cell plasticity through a JNK-dependent mechanism. Oncogene.

[87] Bessede A (2014). Aryl hydrocarbon receptor control of a disease tolerance defence pathway. Nature.

[88] Denison MS, Nagy SR (2003). Activation of the aryl hydrocarbon receptor by structurally diverse exogenous and endogenous chemicals. Annu Rev Pharmacol Toxicol.

[89] Tan Z (2002). Activation of mitogen-activated protein kinases (MAPKs) by aromatic hydrocarbons: role in the regulation of aryl hydrocarbon receptor (AHR) function. Biochem Pharmacol.

[90] Saigusa T, Bell PD (2015). Molecular pathways and therapies in autosomal-dominant polycystic kidney disease. Physiology (Bethesda).

[91] Mandi Y, Vecsei L (2012). The kynurenine system and immunoregulation. J Neural Transm (Vienna).

[92] Sussman CR (2020). Modulation of polycystic kidney disease by G-protein coupled receptors and cyclic AMP signaling. Cell Signal.

[93] Zhang M (2019). Nonselective cyclooxygenase inhibition retards cyst progression in a murine model of autosomal dominant polycystic kidney disease. Int J Med Sci.

[94] Sankaran D (2007). Selective COX-2 inhibition markedly slows disease progression and attenuates altered prostanoid production in Han:SPRD-cy rats with inherited kidney disease. Am J Physiol Renal Physiol.

[95] Salah SM (2019). MCP-1 promotes detrimental cardiac physiology, pulmonary edema, and death in the cpk model of polycystic kidney disease. Am J Physiol Renal Physiol.

[96] Qian F (2012). Effects of 1-methyltryptophan stereoisomers on IDO2 enzyme activity and IDO2-mediated arrest of human T cell proliferation. Cancer Immunol Immunother.

[97] Brincks EL (2020). Indoximod opposes the immunosuppressive effects mediated by IDO and TDO via modulation of AhR function and activation of mTORC1. Oncotarget.

[98] Favennec M (2015). The kynurenine pathway is activated in human obesity and shifted toward kynurenine monooxygenase activation. Obesity (Silver Spring).

[99] Nowak KL (2021). Overweight and obesity and progression of ADPKD. Clin J Am Soc Nephrol.

[100] Heischmann S (2018). Regulation of kynurenine metabolism by a ketogenic diet. J Lipid Res.

[101] Strasser B (2015). Effects of a caloric restriction weight loss diet on tryptophan metabolism and inflammatory biomarkers in overweight adults. Eur J Nutr.

[102] Tang K (2021). Indoleamine 2,3-dioxygenase 1 (IDO1) inhibitors in clinical trials for cancer immunotherapy. J Hematol Oncol.

[103] Prendergast GC (2017). Discovery of IDO1 inhibitors: from bench to bedside. Cancer Res.

[104] Gainullin VG (2015). Polycystin-1 maturation requires polycystin-2 in a dose-dependent manner. J Clin Invest.

[105] Yuan M (2012). A positive/negative ion-switching, targeted mass spectrometry-based metabolomics platform for bodily fluids, cells, and fresh and fixed tissue. Nat Protoc.

[106] Zhu W (2011). Quantitative profiling of tryptophan metabolites in serum, urine, and cell culture supernatants by liquid chromatography-tandem mass spectrometry. Anal Bioanal Chem.

[107] Chong J (2018). MetaboAnalyst 4.0: towards more transparent and integrative metabolomics analysis. Nucleic Acids Res.

