# Supplementary material for: The tryptophan-metabolizing enzyme indoleamine 2,3-dioxygenase 1 regulates polycystic kidney disease progression
Source: JCI Insight. 2023 Jan 10;8(1):e154773. doi: 10.1172/jci.insight.154773 (PMC9870090; doi:10.1172/jci.insight.154773)
Supplement: Supplemental data [file jciinsight-8-154773-s139.pdf]

Supplemental Material

Title: The Tryptophan Metabolizing Enzyme Indoleamine 2,3-Dioxygenase 1 Regulates Polycystic Kidney Disease Progression

Dustin T. Nguyen[1*], BA; Emily K. Kleczko[1*], PhD; Nidhi Dwivedi[1], PhD; Marie-Louise T. Monaghan[1], PhD; Berenice Y. Gitomer[1], PhD; Michel B. Chonchol[1,2], MD; Eric T. Clambey[3], PhD; Raphael A. Nemenoff[1,2], PhD; Jelena Klawitter, PhD[1,3]; Katharina Hopp[1,2], PhD

[1]Department of Medicine, Division of Renal Diseases and Hypertension; [2]Consortium for Fibrosis Research and Translation; [3]Department of Anesthesiology; University of Colorado Anschutz Medical Campus, Aurora, CO 80045, USA *Contributed equally to this work.

Corresponding Author:

Dr. Katharina Hopp

Division of Renal Diseases & Hypertension

University of Colorado Anschutz Medical Campus

12700 E 19th Avenue, P15-7400A, Mail Stop C281

Aurora, CO 80045

Phone: 303-724-1858

Fax: 303-724-4868

E-mail: katharina.hopp@cuanschutz.edu

Supplemental Methods

Experimental study

All animal procedures were performed in an AAALAC-accredited facility in accordance with the *Guide for the Care and Use of Laboratory Animals*(1) and approved by the University of Colorado Anschutz Medical Campus Institutional Animal Care and Use Committee (protocol #33, #685). From birth until 24 days of age mice were housed with their parents. At 24 days of age mice were weaned with same sex/age mice which were part of the same study: 3-5 mice/cage. For the rest of the duration of the study mice were housed in the vivarium, which maintains a temperature of ~72ºF, humidity of ~37%, and light cycle of 8pm off, 6am on. All cages were sterile, and animals received hyperchlorinated reverse osmosis water delivered via an automatic watering system and an irradiated diet (ENVIGO #2920).

Mouse models

Fully inbred, homozygous C57Bl/6J *Pkd1*$^{RC/RC}$ mice were obtained from the Mayo Clinic, Rochester, MN (Dr. Peter C. Harris) in 2015 with an approved MTA and were maintained in homozygosity by the study PI, Katharina Hopp(2, 3). Homozygous C57Bl/6J *Pkd1*$^{RC/RC}$ mice were outcrossed every 10th generation to wildtype C57Bl/6J mice obtained from *The Jackson Laboratory* (stock #000664). The C57Bl/6 Ido1 knock-out (*Ido1*$^{-/-}$, stock #005867) mice were purchased from *The Jackson Laboratory*. For the treatment studies, all C57Bl/6J *Pkd1*$^{RC/RC}$ experimental animals were from an 8th generation backcross and their genotype was confirmed as previously published(3, 4). For the genetic studies, C57Bl/6 *Pkd1*$^{RC/RC}$ and *Ido1*$^{-/-}$ mice were crossed for two generations. All experimental animals (C57Bl/6 *Pkd1*$^{RC/RC}$;*Ido1*$^{+/+}$ *or* C57Bl/6 *Pkd1*$^{RC/RC}$;*Ido1*$^{+/+}$) were F3 animals. All mice were sacrificed at the indicated 3-month and 6-month time points and both sexes, males and females, were utilized for the study. The C57Bl/6J *Pax8*^{rtTA};TetO-cre;*Pkd2*$^{flx/flx}$ model was received from the NIH NIDDK PKD Research Resource

Consortium (University of Maryland, Baltimore, MD, Dr. Terry Watnick; *The Jackson Laboratory*, stock #017292, #007176, #006234)(5-7). *Pkd2* loss was induced by intraperitoneal injection of doxycycline (Sigma, #D9891) dissolved in sterile water at a dose of 40mg/kg at postnatal day (P) 10 and a dose of 50mg/kg at P11. All mice were sacrificed at the indicated time points (P22 for 1-MT study and P16 for Supplemental Figure 6). For the Kaplan-Meier analyses animals were aged until found dead in the cage or until veterinarian requested euthanasia of the animal due to poor health. Both sexes, males and females, were utilized for each study. Wildtype control animals were *Pkd2*$^{flx/flx}$ mice either negative for the TetO-cre or *Pax8*^{rtTA} transgene.

IDO1 inhibition

One-month-old C57Bl/6J *Pkd1*$^{RC/RC}$ mice were treated by oral gavage for three weeks. The following two groups were treated simultaneously: IDO1 inhibitor: twice daily, 400 mg/kg, 1-Methyl-tryptophan (Sigma-Aldrich, #452483), 80mg/ml dissolved in 0.5% hydroxypropyl methyl cellulose, 0.1% Tween 80; Control: 0.5% hydroxypropyl methyl cellulose, 0.1% Tween 80. The dosing regimen and drug concentration for 1-MT delivery via oral gavage has been extensively studied in preclinical cancer and arthritis studies(8-10). Since 1-MT administration via oral gavage in neonates and juvenile mice is not feasible, C57Bl/6J *Pax8*^{rtTA};TetO-cre;*Pkd2*$^{flx/flx}$ mice received daily IP injections of 100mg/kg 1-MT or control starting at P12 until P21. 1-MT was dissolved in 1 N HCl and adjusted to pH 7.0 with sterile PBS and 1 M NaOH before injection. The chosen 1-MT IP dosing regimen and drug concentration have been previously published for use in preclinical mouse models(11-13). Control animals received equal volume of buffered PBS.

Human samples

De-identified ADPKD patient cyst cells were obtained from the Baltimore Polycystic Kidney Disease Research and Clinical Core Center (NIDDK, P30DK090868, University of Maryland, Baltimore, MD, Dr. Terry Watnick;). Each cell colony was established from a single cyst, was

passaged once, and then shipped. For some cysts, multiple primary cell cultures were established.

Mouse tissue harvest/analysis

Animals were euthanized by isoflurane exposure followed by cervical dislocation, and the body weight of each animal was recorded. A terminal blood collection was performed by cardiac puncture. The kidneys were harvested and weighed. The left kidney was used for the single cell suspension/flow cytometry. The right kidney was cut into 2-5mm sections at each pole and the center, fixed in 4% paraformaldehyde, and embedded in paraffin for histological analyses. The remainder of the kidney was flash frozen in liquid nitrogen for follow-up analyses such as metabolomics or western blotting. Of note, four flash frozen kidney samples and terminal blood collections of *Pax8*^{rtTA};TetO-cre;*Pkd2*$^{flx/flx}$ mice that were part of the 1-MT treatment study were lost due to improper storage and could not be analyzed for blood urea nitrogen (BUN) or tryptophan metabolites.

Cell culture

Cell lines (RCTE & 9-12) used have been previously described(14). RCTE cells are *PKD1*$^{+/+}$ immortalized human renal cortical tubular epithelial cells, and 9-12 cells are *PKD1*$^{-/-}$ immortalized cells derived from human ADPKD cystic epithelium. All primary cyst cells and cell lines were grown in Dulbecco’s modified Eagle’s medium/Ham’s F-12 50/50 mix with L-glutamine and 15nM HEPES (DMEM/F12; Corning) supplemented with 10% fetal bovine serum (Sigma-Aldrich) and 1% penicillin-streptomycin (Corning). Cells were grown in a humidified incubator with 5% CO2 at 37°C.

Cell viability assay

10,000 RCTE & 9-12 cells in exponential growth phase were seeded in 96-well plates and a 3-(4,5-dimethylthiazol-2-yl)-2,5-diphenyltetrazolium (MTT, Thermo Fischer Scientific. #M6494) assay was performed. Cells were treated with vehicle (PBS), or 1-methyl-D-tryptophan dissolved in PBS (1-MT, Sigma-Aldrich, #452483) at indicated doses and harvested at indicated times. Following, cells were incubated in MTT solution (5mg/mL) for 1h in 5% CO2 incubator. After incubation, MTT solution was washed off and intracellular purple formazan was solubilized in DMSO. Cells were read in a spectrophotometer at 540nM. Each measurement contains six replicates per cell line, dose, and time point. Spectrometer readings of 1-MT treated cells were normalized to vehicle treated cells set at 100% viability.

IFN-γ stimulation

Cultured RCTE and 9-12 cells were grown to 70% confluence on 10cm culture plates. Human recombinant IFN-γ (PeproTech, Rocky Hill, NJ, #300-02) was added to culture medium at 100 ng/ml for 24 hours. Control cells received phosphate-buffered saline. After 24h, cells were harvested and lysed by scraping down plates with ice cold PBS, centrifuging at 300g for 5 minutes, and incubating the cell pellet in RIPA lysis buffer with 1:100 Protease Inhibitor Cocktail (Sigma-Aldrich, #P8340) for 10 minutes after vortexing. The cells were then centrifuged at 13,000rpm for 5 minutes, and cell lysate supernatant was transferred to clean tubes.

Kidney function analyses

BUN was measured using the QuantiChrom Urea Assay Kit (BioAssay Systems, # 501078333) according to the manufacturer's protocol. Whole blood was collected via cardiac puncture at time of dissection and collected in heparin-coated tubes. Plasma was isolated via centrifugation (1,500xg, 15min). 5 μL of plasma were analyzed per sample and all animals were analyzed in duplicates.

Histomorphometric analysis

Image acquisition and analysis was performed as previously described(4). Cystic index, cyst size, and cyst number were analyzed using a custom-built NIS-Elements AR v4.6 macro (Nikon) using three cross-sections per kidney (two poles and center/pelvis). A cyst was defined as having a minimum feret diameter of 50µm. The cystic index was defined as the percentage of cystic area per kidney cross section, and cyst number was normalized to area. Fibrotic area was analyzed from picrosirius red stained kidney sections and visualized using an Olympus BX41 microscope with a linear polarizer. Ten random cortical 40x images were analyzed per animal and the percent fibrotic area was calculated from total kidney tissue area. Fibrillar collagen (birefringent area) was quantified using ImageJ. Computed volumes were calculated by multiplying the obtained indices (cystic or fibrotic) with the kidney weight of the same animal.

Immunofluorescence labeling/quantification

Tissues were prepared for immunofluorescence labeling as previously described(4). Briefly, 4µm sections underwent autofluorescence quenching with 0.1% Sudan Black in 70% ethanol, 2h citrate antigen retrieval at 100ºC, and washes in TBST. Primary antibodies used were anti-mouse IDO1 (clone mIDO-48; BioLegend #122402) at 1:50 and anti-mouse E-cadherin (clone 36; BD Transduction Laboratories #610182) at 1:100. Secondary antibodies used were AF488 goat anti-rat IgG, AF594 goat anti-rabbit IgG, and AF647/AF488 goat anti-mouse IgG2a (Life Technologies) at 1:1000. Slides were mounted using VectraShield (Vector Laboratories, #H-1200). Image visualization was performed using a Nikon *Eclipse Ti* microscope with a Zyla 4.2sCMOS camera. Image analysis was done using NIS-Elements AR v4.6 (Nikon).

Western blotting

Kidney tissue was homogenized in lysis buffer containing RIPA and Protease Inhibitor Cocktail (Sigma Aldrich, #P8340) using a Qiagen TissueLyser LT homogenizer. Protein concentration was

measured using Protein Assay Dye Reagent Concentrate (Bio-Rad, #5000006) and 30µg of samples were loaded onto 4-12% acrylamide gels and ran for 1 hour at 200 V. Cultured cell lysates and human cyst lysates were loaded at 30µg protein per well. Gels were transferred to PVDF membranes at 400 mAmps for 3 hours followed by blocking in 5% BSA. The primary antibodies used were IDO1 (clone mIDO-48; BioLegend, #122402, 1:400), IDO2 (clone 1HC; Novus Biologicals, #NBP2-21641, 1:500), Kynureninase/KYNU (clone 771312; R&D Systems #MAB7389, 1:500), Kynurenine 3-Monooxygensase/KMO (clone 2493A; R&D Systems #MAB8050-SP, 1:500), Kynurenine aminotransferase/KAT (clone C-7; Santa Cruz Biotechnology, #sc-374531, 1:500), GAPDH (polyclonal FL-335, Santa Cruz Biotechnology, # sc-25778, 1:500). Respective secondary antibodies (anti-rat-HRP [1:5,000], secondary anti-mouse-HRP [1:10,000], and secondary anti-rabbit-HRP [1:5,000], all Jackson ImmunoResearch, West Grove, PA) were used. Primary antibodies were prepared in 5% nonfat dry milk and membranes incubated overnight with gentle agitation in a 4°C refrigerator. Secondary antibodies were prepared in 5% BSA and membranes incubated for 1h at room temperature. Membranes were washed three times in 1X TBST and exposed to Western Lightning Plus-ECL substrate (PerkinElmer, #NEL104001EA). Blots were developed by chemiluminescence and densitometry on X-ray films was quantified using ImageJ software.

Single cell suspension

Dissected kidney tissue was mechanically dissociated using razor blades and placed in 3.6mL of DMEM/F12 media (Corning) with 0.4mL Liberase TL (2mg/mL in DMEM/F12, Sigma-Aldrich, #05401020001), and 20µL DNaseI (20K U/mL in Hank's Buffer; Sigma-Aldrich, #D5025). Tissue suspensions were placed in a 37°C shaking water bath for 30 minutes, and the samples were mixed every 10 minutes. To ensure dissociation, the digestion mix was passed through an 18G needle several times. An equal volume of FA3 Buffer (PBS, 10mM HEPES [Corning], 2nM EDTA, 1% FBS [Sigma-Aldrich]) was added to the tissue digestion mixture and strained through a 100

μm filter. Single cells were spun down and washed in FA3 Buffer. The cell pellet was then resuspended in 2 mL of Red Blood Cell (RBC) Lysis Buffer (0.015M NH_4Cl, 10mM $KHCO_3$, 0.1mM Na_2EDTA, pH 7.2) for exactly 3 minutes at room temperature. The lysis was quenched by adding 13mL of FA3 Buffer. After centrifuging, the cell pellet was resuspended in 10 mL of FA3 Buffer and passed through a 70 μm filter. The resulting pelleted cells were then ready for flow cytometry staining.

Flow cytometry

The single cell suspension was blocked in anti-mouse CD16/CD32 (clone 93; eBioscience, #14-0161-86) at 1:200 on a rocker for 15 min at 4°C. Following, viability dye (LIVE/DEAD Fixable Aqua Dead Cell Stain Kit, Invitrogen, #L34966) was added and the suspension was stained for 15 min at 4 °C. The cells were then washed once with FA3 and then split in half for staining of two different flow cytometry panels.

<u>Panel 1</u>

To one half of the single cell suspension the following conjugated antibodies to surface markers were added: CD45-FITC (clone 30-F11; 1:100; BioLegend), CD11c-PE (clone HL3; 1:100; BD Biosciences), F4/80-PE/Dazzle594 (clone BM8; 1:100; BioLegend), CD11b-PerCP-Cy5.5 (clone M1/70; 1:100; BD Biosciences), Gr-1-PE/Cy7 (clone RB6-8C5; 1:100; BioLegend), PD-L1-APC (clone MIH5; 1:100; eBioscience), MHCII-DyLight680 (clone M5/114.15.2; 1:100; Novus), EpCAM-APC-eFluor780 (clone G8.8; 1:100; eBioscience), NKp46-eFluor450 (clone 29A1.4; 1:100; eBioscience), and CD4-V500 (clone RM4-5; 1:100; BD Biosciences; used only for compensation). Cells were incubated with all antibodies in the dark on a rocker at 4°C for 60 min followed by two washes in FA3 buffer. Finally, cells were resuspended in FA3 Buffer and ran on the Gallios Flow Cytometer Machine (Beckman Coulter). For compensation, single stained beads (VersaComp Antibody Capture Bead Kit; Beckman Coulter) and a cell mix of all samples were used.

Panel 2

To the second half of the single cell suspension the following conjugated antibodies were added on day 1: CD44-FITC (clone IM7; 1:100; eBioscience), PD-1-PE (clone RMP1-30; 1:100; BD Biosciences), CD45-PE-CF594 (clone 30-F11; 1:100; eBioscience), TCRβ-PE-Cyanine5 (clone H57-597; 1:100; eBioscience), CD69-PE-Cy7 (clone H1.2F3; 1:100; eBioscience), CD8-Alexa Fluor 700 (clone 53-6.7; 1:100; eBioscience), CD4-APC/Cyanine7 (clone GK1.5; 1:100; BioLegend), and CD4-V500 (clone RM4-5; 1:100; BD Biosciences; used only for compensation). Cells were incubated with the antibody mix in the dark on a rocker at 4°C for 60 min. Following, cells were washed with FA3 buffer and then incubated in fixation/permeabilization buffer overnight according to the Foxp3/Transcription Factor Staining Buffer Set (eBioscience, #00-5523-00). The next day, conjugated antibodies with intracellular targets were added to cells in permeabilization buffer (Ki-67-APC [clone SolA15; 1:400; eBioscience] and FoxP3-eFluor450 [clone FJK-16s; 1:200; eBioscience]) and cells were incubated with the antibody mix for 120 minutes on a rocker at 4°C in the dark. Cells were washed twice with permeabilization buffer, resuspended in FA3 buffer and ran on the Gallios Flow Cytometer Machine (Beckman Coulter). Compensation using single stained bead and a pooled cell mix was performed.

Data analysis

Flow cytometry data were analyzed using the Kaluza Analysis v2.1 software (Beckman Coulter). First, compensation for each channel was performed using single-stained beads and confirmed using single stained pooled cell mix. All samples were then analyzed using the gated workflow shown in **Supplemental Figure 9** in a blinded manner.

Metabolomics - Liquid chromatography tandem mass spectrometry (LC/MS-MS)

Semi-quantitative targeted metabolomics

Sample analysis was performed based on a validated approach(15, 16). Kidneys were perfused with ice cold PBS/heparin, and kidneys were dissected and snap frozen in liquid nitrogen.

Following, kidney tissue samples (~50-100 mg) were homogenized in an adequate volume of 80% (v/v) cooled methanol, incubated for protein precipitation, dried in a SpeedVac concentrator centrifuge (Savant, ThermoFisher), and reconstituted in water/methanol. 8 µL of sample was injected onto an Amide XBridge HPLC column (3.5 µm; 4.6 mm inner diameter (i.d.) × 100 mm length; Waters). The mobile phases consisted of HPLC buffer A (pH = 9.0: 95% [vol/vol] water, 5% [vol/vol] acetonitrile, 20 mM ammonium hydroxide, 20 mM ammonium acetate) and HPLC buffer B (100% acetonitrile). The HPLC settings were as follows: from 0 to 3 minutes, the mobile phase was kept at 85% B; from 3 to 22 minutes, the percentage of solvent B was decreased from 85% to 2% and was kept at 2% for an additional 3 minutes. At minute 26, solvent B was increased again back to 85% and the column flushed for an additional 7 minutes at 85% solvent B.

The Q1 (precursor ion) and Q3 (fragment ion) transitions, the metabolite names, dwell times and the appropriate collision energies (CEs) for both positive and negative ion modes were adapted from(15) with several additional transitions. Q1 and Q3 transitions were set to unit resolution for optimal metabolite ion isolation and selectivity. In addition, the polarity switching (settling) time was set to 50 ms; in 1.42 s using a 3-ms dwell time, we were able to obtain 6-14 scans per metabolite peak. The source temperature was set at 500°C, curtain gas (CUR, nitrogen) at 20, collision gas (CAD, nitrogen) at high, ion source gases 1 and 2 at 33, declustering potential (DP) at +93/-93, entrance potential (EP) at +10/-10, and collision cell exit potential (CXP) at +10/-10 for positive and negative ion modes, respectively. Positive identification of the metabolites of interest was performed through injection of pure compound standards onto the above-described LC-MS/MS platform (confirmation of the fragmentation pattern [MS/MS] and retention time).

Kynurenines

Kynurenines were analyzed using a modification of(17). Briefly, frozen tissue (~15 mg) was weighed and homogenized in 0.5 mL formic acid (10% in water) / methanol (30/70, v/v) using an electric homogenizer. The extraction solution was enriched with isotope labeled internal standard mix (at 10 ng/mL final concentration, see below). Samples were vortexed and centrifuged at

26,000xg for 20 minutes after which the supernatant was transferred into HPLC vials with glass inserts. Calibrator standards and quality control samples were prepared in 0.1% formic acid in water as surrogate matrix. LC-MS/MS was performed on an Agilent Technologies 1200 HPLC system connected to an ABSCIEX 5500 QTRAP mass spectrometer equipped with a turbo ion spray source operated in electrospray mode. LC separation was carried out on an Atlantis T3 3µm (2.1x50 mm) column (Waters Corp.) using a mobile phase consisting of 0.1% formic acid in water (Solvent A) and acetonitrile (Solvent B). All analytes were detected in positive ion multiple reaction monitoring (MRM) mode. The following quantifier ion-transitions were monitored TRP (205>118), KYN (209>192), KYNA (190>144), 3OH KYN (225>208), anthranilic acid (AA) (138>120), picolinic acid (PA) (124>78), QA (168>78), 3-OH AA (154>80), and serotonin (SER) (177>115). The following isotope labeled internal standards were used: d_5-TRP (210.2>147.2), $^{13}C_3$,^{15}N-3OH KYN (229.2>110.2), d_5-KYNA (195.2>149.2), $^{13}C_4$,^{15}N-QA (173>81.2), $^{13}C_6$-KYN (215.2>152.2), $^{13}C^6$-AA (144>98.2), d_4-PA (128>82.2), d_4-SER (181>164).

Data analysis

MetaboAnalyst 4.0 (University of Alberta, Canada) was used for statistical analysis of metabolomics data(18). Relative peak intensities were initially normalized to the deuterated internal standards followed by the sum of all integrals and tissue weight. After that, data were log transformed and then Paretto-scaled (mean centered and divided by the square root of the SD of each variable). ANOVA with post-hoc Tukey HSD was used to compare group differences. Analysis of changes in metabolites between different animal groups was performed by utilizing Partial Least Squares-Discriminant Analysis (PLS-DA). False discovery rate (FDR) correction was applied to correct for multiple comparisons (FDR < 0.05 for statistical significance).

Statistical analysis

All analyses were performed using PRISM9 (Graphpad Software). Data are depicted as mean ± standard error of the mean (SEM) or box plot with whiskers of 10-90th percentile; single data points

are depicted in all instances. Analyses were performed as unpaired t test, or one-way ANOVA with Tukey's multiple comparison test (including ROUT [Q=1%] outlier testing), depending on data type and group number. P-values are denoted by *($P<0.05$), **($P<0.01$), ***($P<0.001$), and ****($P<0.0001$).

Supplemental Figures

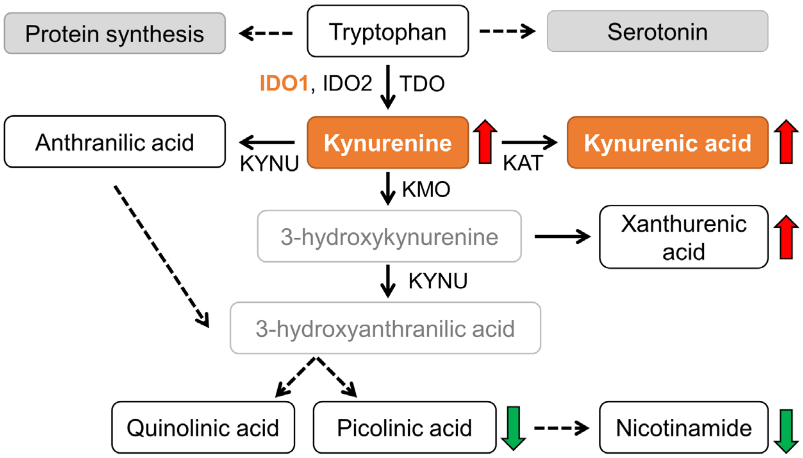

Supplemental Figure 1 | Schematic of tryptophan metabolism. Key catabolites analyzed by mass spectrometry are depicted (black/orange; Orange indicates catabolites or enzymes known to have immunomodulatory roles. Colored arrows indicate trend of levels in C57Bl/6J *Pkd1*$^{RC/RC}$ kidneys versus wildtype (red: increased, green: decreased). IDO: indoleamine 2,3-dioxygenase; TDO: tryptophan 2,3-dioxygenase; KATs: kynurenine amino transferases; KMO: kynurenine 3-monooxygenase; KYNU: kynureninase.

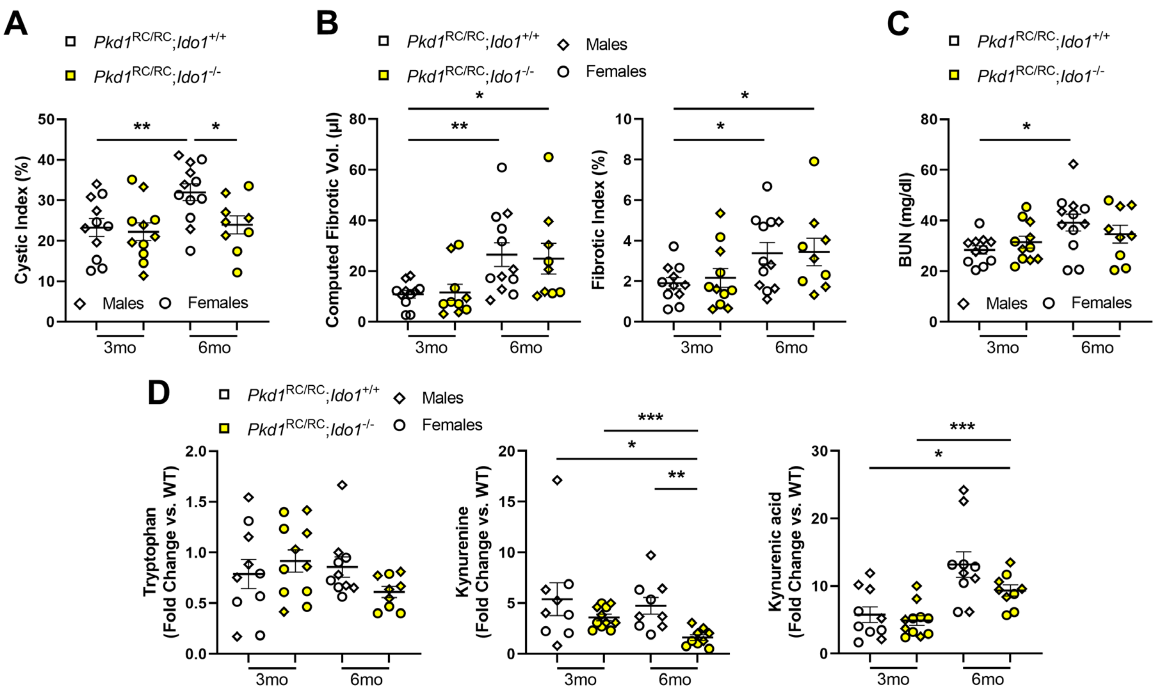

Supplemental Figure 2 | PKD-related histopathological quantification and metabolomics highlight reduced PKD severity and correction of dysregulated tryptophan metabolism in PKD mice null for *Ido1* versus control. Quantification of (A) cystic index (B) fibrosis and (C) BUN highlight reduction in cystic kidney disease severity but not fibrotic burden or kidney function decline in *Pkd1*$^{RC/RC}$; *Ido1*$^{+/+}$ (white) versus *Pkd1*$^{RC/RC}$; *Ido1*$^{-/-}$ (yellow) animals. (D) Levels of tryptophan metabolites of kidneys from *Pkd1*$^{RC/RC}$; *Ido1*$^{+/+}$ (white) and *Pkd1*$^{RC/RC}$; *Ido1*$^{-/-}$ (yellow) animals quantified as fold-change compared to genotype-, age-, and gender- matched wildtype (WT) mice. At 3mo and 6mo of age, levels of the immunosuppressive tryptophan metabolite kynurenine are significantly reduced in PKD *Ido1* null animals compared to control, reaching close to WT levels. At 6mo of age, the time point at which a significant reduction in PKD severity is observed in PKD *Ido1* null animals versus control, levels of kynurenic acid also declined, although not significant. N= 5males (diamond), 4-7females (circle) per genotype and time point. Statistics: Graphs: mean ± SEM; Analyses: one-way ANOVA with Tukey's multiple comparison test. P-values *<0.05, **<0.01, ***<0.001, comparisons with non-significant statistics are not shown.

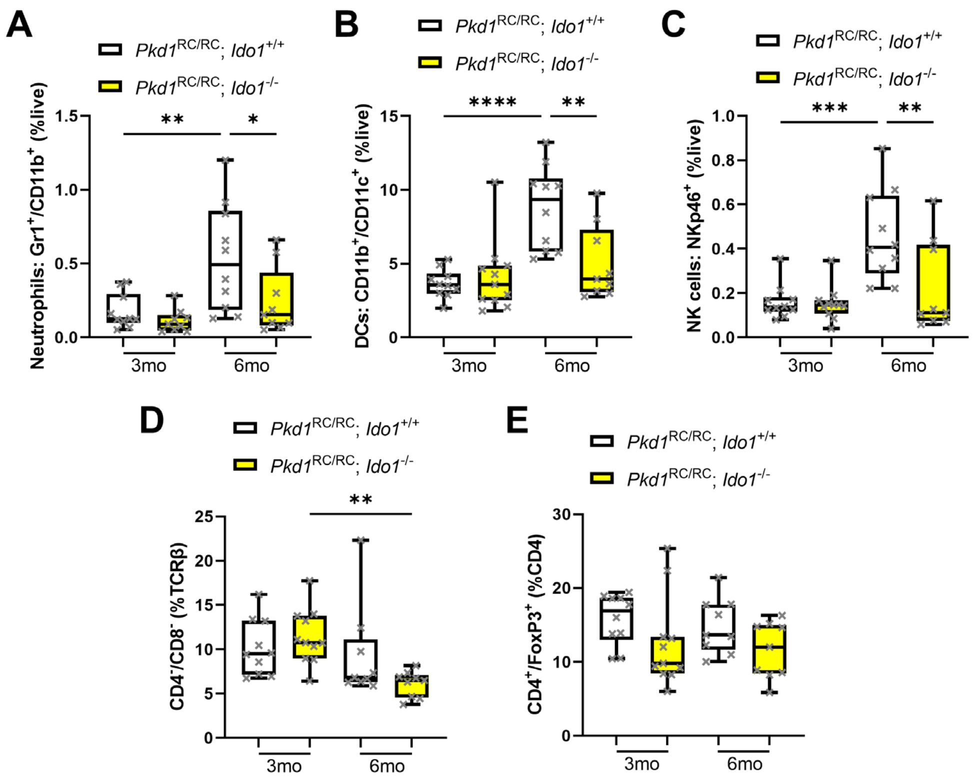

Supplemental Figure 3 | Kidney innate and adaptive immune cell numbers are altered in PKD *Ido1* null mice compared to control. Quantification of flow cytometry data obtained from kidney single cell suspensions of *Pkd1*$^{RC/RC}$; *Ido1*$^{+/+}$ (white) and *Pkd1*$^{RC/RC}$; *Ido1*$^{-/-}$ (yellow) animals highlights significantly reduced numbers of (A) neutrophils, (B) dendritic cells, and (C) natural killer cells at 6mo of age, the time point at which reduced PKD severity was observed in animals null for *Ido1*. Further, numbers of (D) double negative T cells as percent of all T cells (TCRβ^{+}) were significantly reduced at 6mo and (E) T$_{Regs}$ trend towards a reduction, although not significant, in PKD animals null for *Ido1* versus control at 6mo of age. Statistics: Graphs: box plot, whiskers 10-90th percentile; Analyses: one-way ANOVA with Tukey's multiple comparison test. P-values *<0.05, **<0.01, ***<0.001, ****<0.0001, comparisons with non-significant statistics are not shown. N= 5males, 4-7females per genotype and time point.

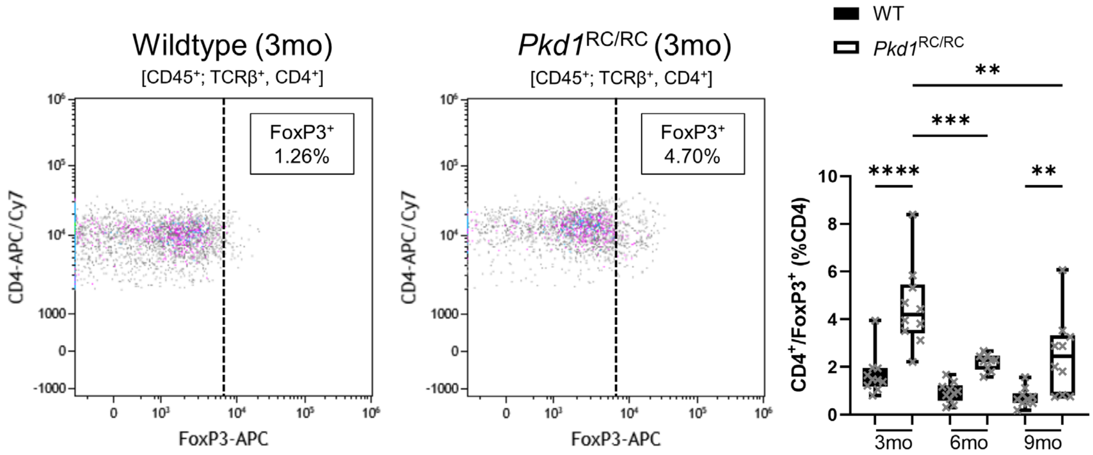

Supplemental Figure 4 | Kidney regulatory T cell numbers increase in *Pkd1*$^{RC/RC}$ mice compared to wildtype. Representative flow cytometry plot indicating the gating strategy of regulatory T cells (CD4$^+$/Foxp3$^+$) in kidney single cell suspensions from C57Bl/6J *Pkd1*$^{RC/RC}$ mice and strain, age, and gender matched wildtype (WT) mice (left). Quantification at 3mo, 6mo, and 9mo (right). Numbers of T_{Regs} are increased at all investigated time points, however the increase is most prominent at milder disease stages (3mo). Statistics: Graphs: box plot, whiskers 10-90th percentile; Analyses: one-way ANOVA with Tukey's multiple comparison test. P-values **<0.01, ***<0.001, ****<0.0001, comparisons with non-significant statistics are not shown. N= 5males, 5females per genotype and time point.

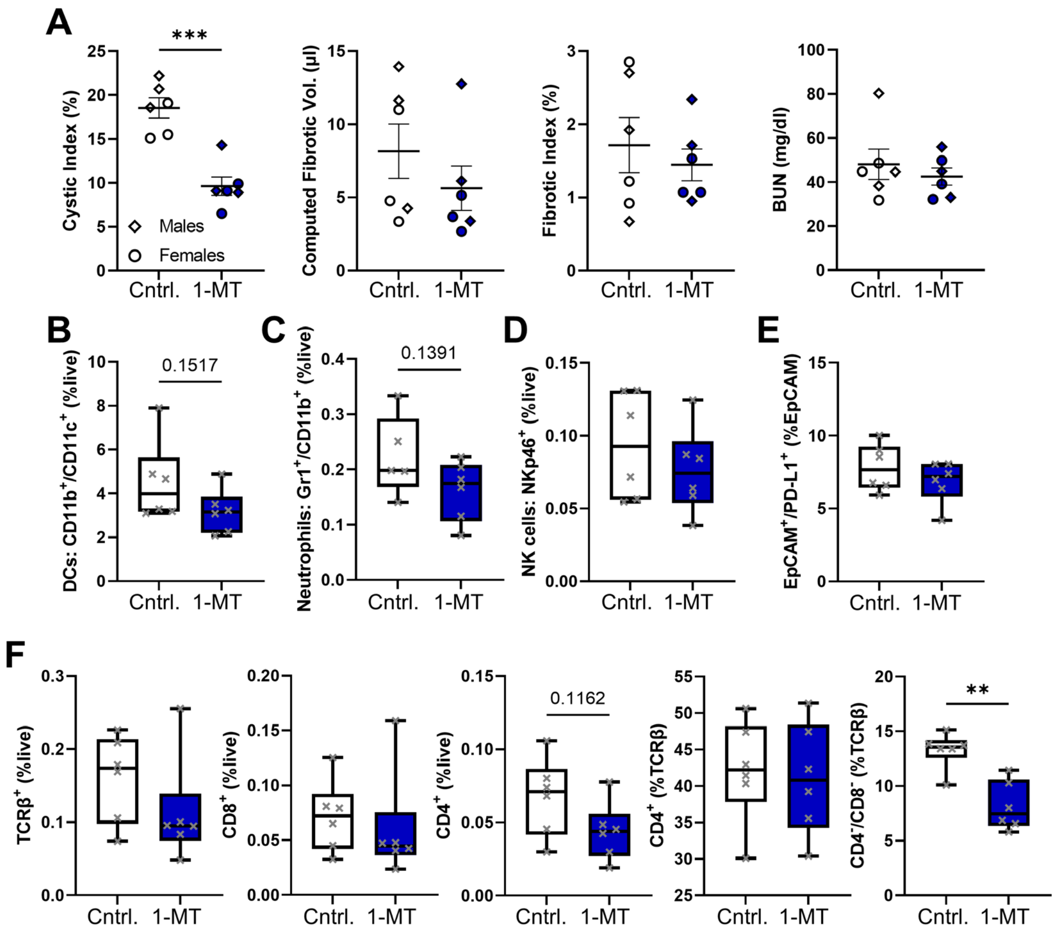

Supplemental Figure 5 | 1-MT treatment in *Pkd1*$^{RC/RC}$ mice results in less severe cystic disease and associates with changes to the innate and adaptive immune microenvironment. Quantification of (A) cystic index, fibrotic volume, and fibrotic index as well as blood urea nitrogen (BUN) levels in *Pkd1*$^{RC/RC}$ mice treated with (blue) or without (white, Cntrl.) 1-MT. While cystic index was significantly reduced upon treatment, the level of fibrosis or kidney function was not altered. Quantification of flow cytometry data obtained from kidney single cell suspensions of *Pkd1*$^{RC/RC}$ animals treated with (blue) or without (white, Cntrl.) 1-MT highlights a trend towards reduced numbers of (B) dendritic cells and (C) neutrophils but not (D) natural killer cells. (E) Expression of the immune checkpoint ligand PD-L1 on *Pkd1*$^{RC/RC}$ EpCAM$^+$ cells trends towards a reduction in treated (blue) versus untreated (white, Cntrl.) mice. (F) Overall T cell numbers (TCRβ$^+$) or their subtypes did not change significantly within *Pkd1*$^{RC/RC}$ kidneys of treated

(blue) versus untreated (white, Cntrl.) mice, but the percent of double negative T cells in respect to all T cells did decrease significantly. N= 3males/3females per treatment group. Statistics: Graphs: (A) mean ± SEM, (B-F) box plot, whiskers 10-90th percentile; Analyses: unpaired t test. P-values **<0.01, ***<0.001, comparisons with non-significant statistics are not shown.

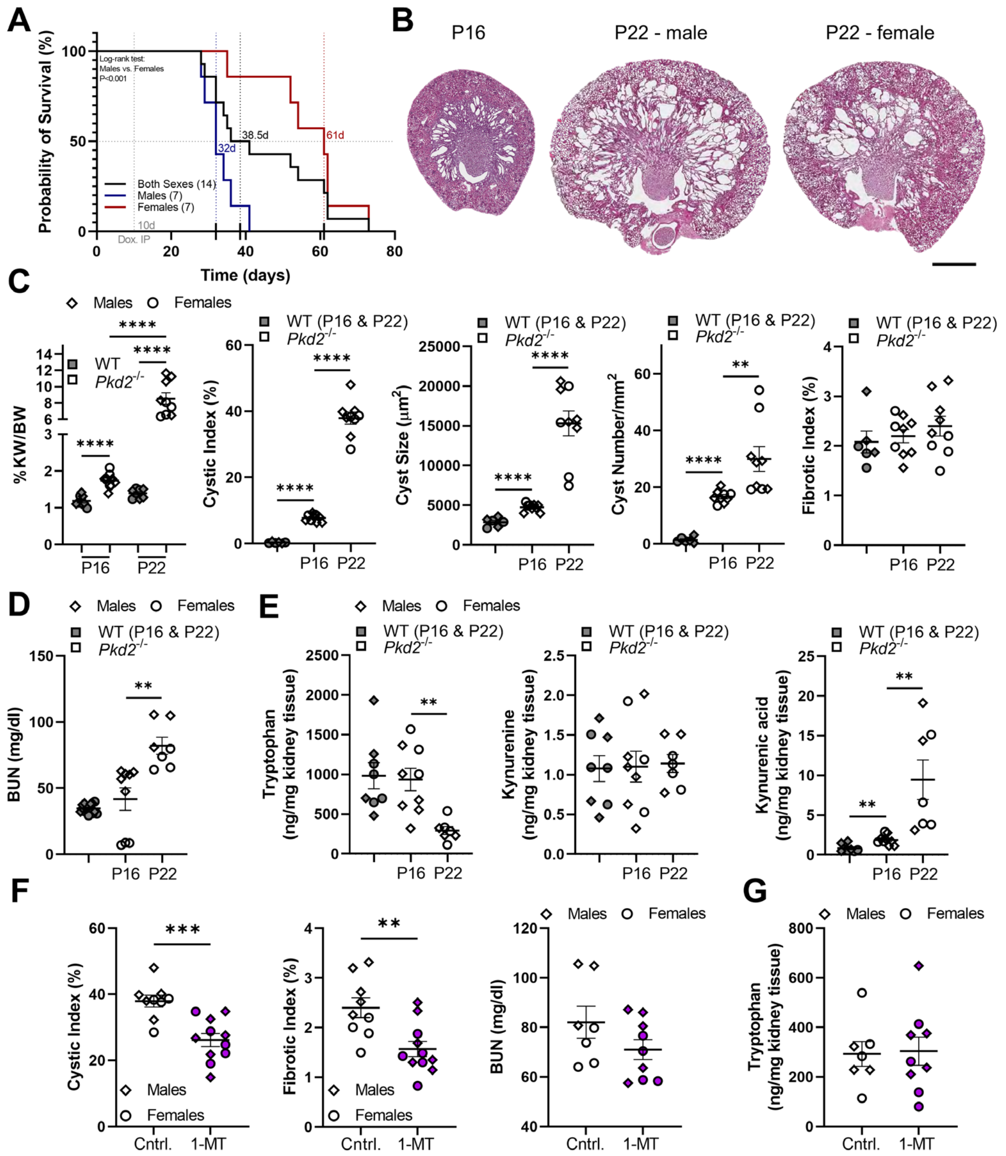

Supplemental Figure 6 | Juvenile induced *Pax8*^{rtTA};TetO-cre;*Pkd2*$^{flx/flx}$ mice present with rapidly progressive PKD marked by dysregulated tryptophan metabolism. (A) Kaplan-Meier survival curve. The 50% survival of *Pax8*^{rtTA};TetO-cre;*Pkd2*$^{flx/flx}$ (*Pkd2*$^{-/-}$)mice is 38.5 days (d); ~28d post induction of *Pkd2* loss via doxycycline IP. Female mice compared to male mice survive longer (50% survival: 61d [females] vs. 32d [males]), which correlates with milder PKD. (B) H&E stained kidney cross sections of *Pax8*^{rtTA};TetO-cre;*Pkd2*$^{flx/flx}$ mice at postnatal (P)16 and P22. Sex dimorphism was not acknowledgeable at P16, hence only one kidney cross section is shown. Quantification of different (C) histological PKD parameters and (D) BUN of *Pax8*^{rtTA};TetO-

cre;*Pkd2*$^{flx/flx}$ mice at P16 & P22 compared to wildtype (WT). For cystic/fibrotic index, cyst size-, and number as well as BUN P16 and P22 wildtype animals were combined as these parameters were comparable between the two ages. (E) Quantification of tryptophan catabolites assayed via mass spectrometry. As noted by significantly elevated kidney kynurenic acid levels in *Pax8*^{rtTA};TetO-cre;*Pkd2*$^{flx/flx}$ mice versus wildtype, the tryptophan pathway is also dysregulated in this rapidly progressive PKD2 model. Scale bars: 1mm, N=3-6males (diamond), 3-6females (circle). Quantification of (F) cystic/fibrotic index and BUN as well as (G) kidney tryptophan metabolite levels in *Pax8*^{rtTA};TetO-cre;*Pkd2*$^{flx/flx}$ mice treated with (purple) or without (white) 1-MT. Treatment (1-MT): P12-21, N=5-6males (diamond), 4-5females (circle). Control: N=3-5males (diamond), 4females (circle). Statistics: mean ± SEM; Analyses: unpaired t test. P-values **<0.01, ***<0.001, ****<0.0001, comparisons with non-significant statistics are not shown.

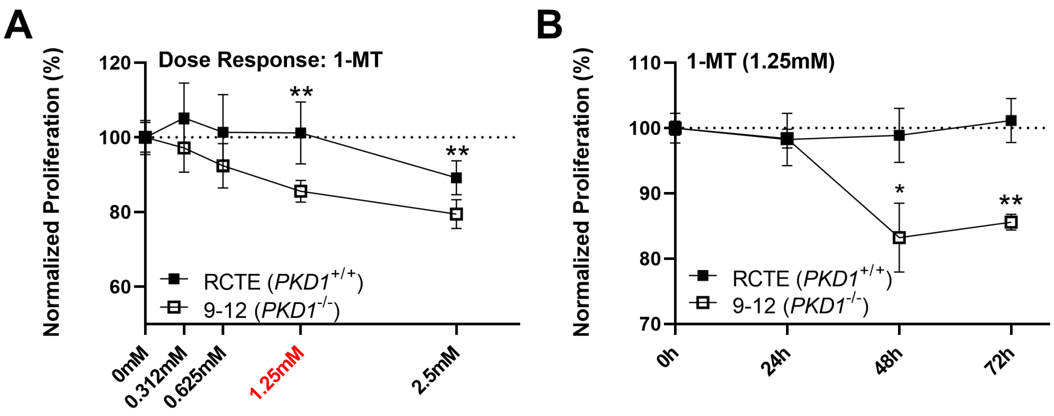

Supplemental Figure 7 | Treatment with 1-MT slows proliferation of ADPKD cells. (A) MTT assay performed on exponentially growing RCTE and 9-12 cells after 72h of treatment with different doses of 1-MT. A 1-MT dose of 1.25mM significantly slowed proliferation of 9-12 but not RCTE cells. (B) Time course of RCTE and 9-12 cells treated with 1.25mM 1-MT and analyzed via MTT assay. Significant differences in rate of proliferation between 9-12 and RCTE cells were observed as early as 48h post treatment start. Proliferation rates are normalized to vehicle (PBS) treated cells. N=6 replicates per cell line/dose/timepoint. Statistics: mean ± SEM; unpaired t test. P-values *<0.05, **<0.01, comparisons with non-significant statistics are not shown.

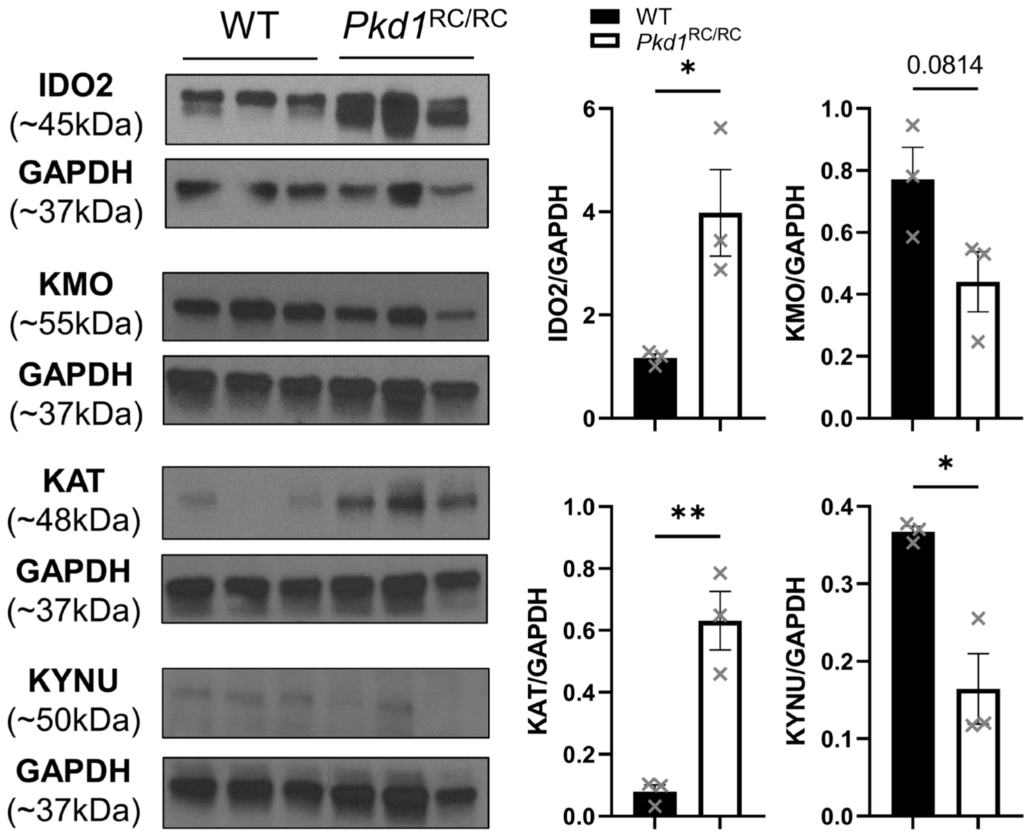

Supplemental Figure 8 | Multiple enzymes in the tryptophan catalysis pathway are dysregulated in PKD. Western blot probing for IDO2, KMO, KAT, and KYNU (left, see Supplemental Figure 1) and quantification (right) using wildtype (WT) and *Pkd1*$^{RC/RC}$ kidney homogenates, N= 3 [male or female], 9mo. IDO2 and KAT levels were increased while KMO and KYNU levels trend towards being decreased in in *Pkd1*$^{RC/RC}$ kidneys compared to WT, all contributing to an enrichment of kynurenine and kynurenic acid in PKD kidneys. IDO: indoleamine 2,3-dioxygenase; KAT: kynurenine amino transferases; KMO: kynurenine 3-monooxygenase; KYNU: kynureninase. Statistics: mean ± SEM; Analyses: unpaired t test. P-value *<0.05, **<0.01.

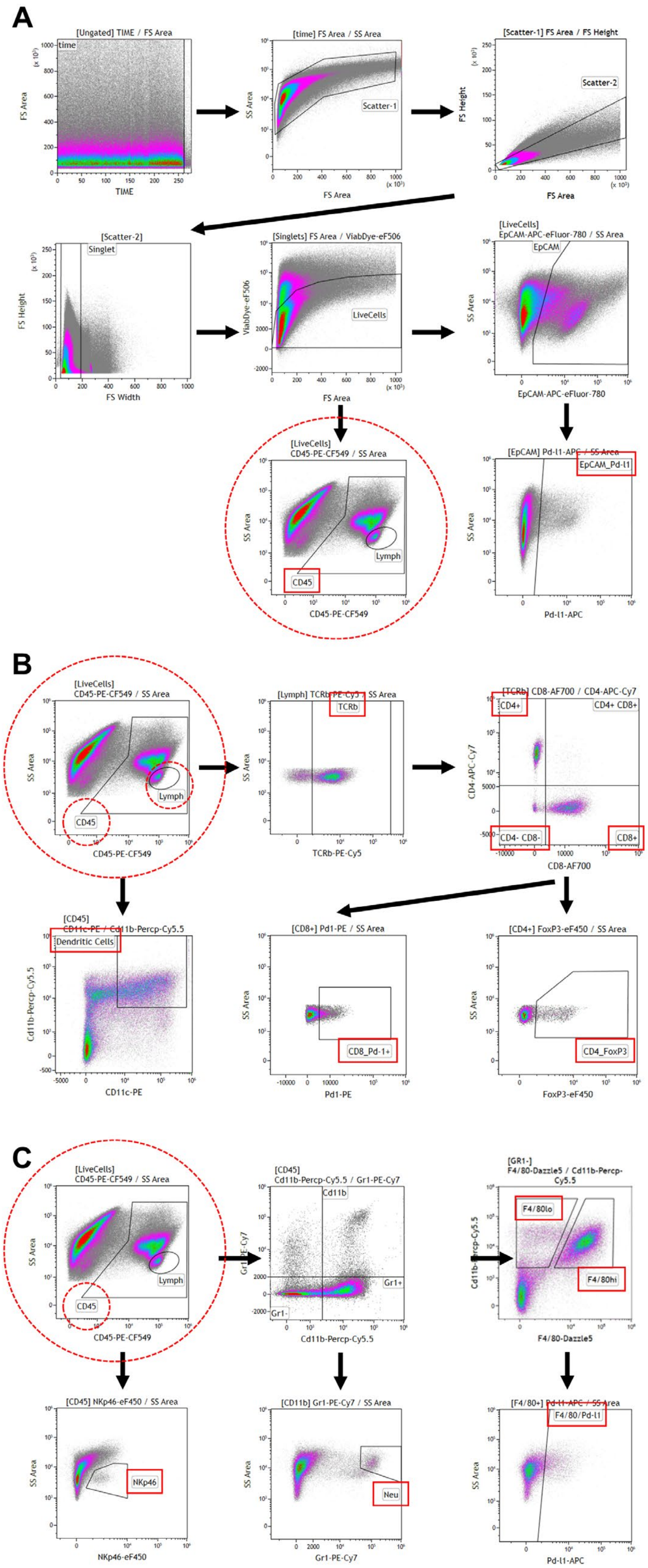

Supplemental Figure 9 | Gating strategy for flow cytometry analysis of all analyzed cellular subtypes. Representative flow images are from 3- and 6-month-old C57Bl/6J *Pkd1*$^{RC/RC}$ mice, taken from three different mice. (A) Gating strategy to identify live, CD45^{+}, singlets and EpCAM^{+};PD-L1^{+} live singlets. (B) Gating strategy identifying different T cell subtypes and CD8^{+};PD-1^{+} expression, starting with a lymphocyte size exclusion gate, as well as CD45^{+};CD11b^{+};CD11c^{+} dendritic cells starting with the CD45^{+} gate. (C) Gating strategy identifying different innate immune cell populations and their PD-L1^{+} expression starting with the CD45^{+} gate.

Supplemental Table 1 | Tryptophan metabolite levels of wildtype and *Pkd1*$^{RC/RC}$ mice separated by sex.

	Metabolite (Abundance/mg tissue [normalized])					
**Sex: Males**	**Tryptophan**	**Kynurenine**	**Kynurenic acid**	**Xanthurenic acid**	**Picolinic acid**	**Nicotinamide**
**3 months**						
WT	$2.02\times10^{-2}\pm0.786\times10^{-2}$	$3.19\times10^{-4}\pm2.02\times10^{-4}$	$1.16\times10^{-2}\pm0.650\times10^{-2}$	$2.99\times10^{-2}\pm0.512\times10^{-2}$	$5.77\times10^{-3}\pm2.48\times10^{-3}$	3.38±0.811
Pkd1$^{RC/RC}$	$2.01\times10^{-2}\pm1.14\times10^{-2}$	$13.2\times10^{-4}\pm12.1\times10^{-4}$	$8.57\times10^{-2}\pm4.43\times10^{-2}$*	$26.6\times10^{-2}\pm19.3\times10^{-2}$*	$4.91\times10^{-3}\pm1.81\times10^{-3}$	3.00±0.713
Fold change	0.995	4.14	7.39	8.9	0.851	0.888
**6 months**						
WT	$2.35\times10^{-2}\pm0.824\times10^{-2}$	$2.96\times10^{-4}\pm1.56\times10^{-4}$	$0.664\times10^{-2}\pm0.180\times10^{-2}$	$4.87\times10^{-2}\pm2.86\times10^{-2}$	$5.37\times10^{-3}\pm1.04\times10^{-3}$	2.94±0.634
Pkd1$^{RC/RC}$	$2.25\times10^{-2}\pm0.993\times10^{-2}$	$15.5\times10^{-4}\pm8.44\times10^{-4}$	$11.2\times10^{-2}\pm5.74\times10^{-2}$***	$26.5\times10^{-2}\pm11.0\times10^{-2}$*	$3.46\times10^{-3}\pm0.373\times10^{-3}$	2.08±0.339* $^{\#}$
Fold change	0.957	5.24	16.9	5.45	0.644	0.707
**9 months**						
WT	$1.93\times10^{-2}\pm0.554\times10^{-2}$	$2.49\times10^{-4}\pm2.84\times10^{-4}$	$0.718\times10^{-2}\pm0.527\times10^{-2}$	$1.43\times10^{-2}\pm0.693\times10^{-2}$	$7.87\times10^{-3}\pm2.52\times10^{-3}$	3.69±1.25
Pkd1$^{RC/RC}$	$1.67\times10^{-2}\pm0.388\times10^{-2}$	$23.5\times10^{-4}\pm10.7\times10^{-4}$**	$8.95\times10^{-2}\pm4.25\times10^{-2}$*	$30.0\times10^{-2}\pm13.4\times10^{-2}$**	$4.53\times10^{-3}\pm1.29\times10^{-3}$	2.92±0.994
Fold change	0.865	9.44	12.5	21.0	0.576	0.791

**Sex: Females**	**Tryptophan**	**Kynurenine**	**Kynurenic acid**	**Xanthurenic acid**	**Picolinic acid**	**Nicotinamide**
**3 months**						
WT	$2.45\times10^{-2}\pm0.874\times10^{-2}$	$1.69\times10^{-4}\pm0.939\times10^{-4}$	$1.04\times10^{-2}\pm0.378\times10^{-2}$	$2.85\times10^{-2}\pm1.02\times10^{-2}$	$7.78\times10^{-3}\pm1.13\times10^{-3}$	3.34±0.998
Pkd1$^{RC/RC}$	$1.51\times10^{-2}\pm0.934\times10^{-2}$	$7.37\times10^{-4}\pm4.39\times10^{-4}$	$4.11\times10^{-2}\pm1.84\times10^{-2}$*	$8.42\times10^{-2}\pm4.43\times10^{-2}$*	$4.48\times10^{-3}\pm3.34\times10^{-3}$	2.56±1.70
Fold change	0.616	4.36	3.95	2.95	0.576	0.767
**6 months**						
WT	$2.37\times10^{-2}\pm0.786\times10^{-2}$	$2.94\times10^{-4}\pm2.10\times10^{-4}$	$0.825\times10^{-2}\pm0.405\times10^{-2}$	$1.64\times10^{-2}\pm0.465\times10^{-2}$	$6.77\times10^{-3}\pm0.965\times10^{-3}$	3.98±1.13
Pkd1$^{RC/RC}$	$1.79\times10^{-2}\pm0.385\times10^{-2}$	$21.5\times10^{-4}\pm21.7\times10^{-4}$	$8.21\times10^{-2}\pm2.22\times10^{-2}$**** $^{\#\#}$	$12.7\times10^{-2}\pm2.98\times10^{-2}$****	$3.01\times10^{-3}\pm0.864\times10^{-3}$	2.06±0.618*
Fold change	0.755	7.31	9.95	7.74	0.445	0.518
**9 months**						
WT	$1.95\times10^{-2}\pm0.764\times10^{-2}$	$3.25\times10^{-4}\pm2.22\times10^{-4}$	$0.658\times10^{-2}\pm0.173\times10^{-2}$	$1.75\times10^{-2}\pm0.515\times10^{-2}$	$7.27\times10^{-3}\pm2.01\times10^{-3}$	3.52±1.33
Pkd1$^{RC/RC}$	$1.80\times10^{-2}\pm0.867\times10^{-2}$	$19.2\times10^{-4}\pm14.4\times10^{-4}$	$8.10\times10^{-2}\pm1.96\times10^{-2}$**** $^{\#\#}$	$14.6\times10^{-2}\pm3.85\times10^{-2}$**** $^{\#}$	$3.73\times10^{-3}\pm2.68\times10^{-3}$	2.17±1.36
Fold change	0.923	5.91	12.3	8.34	0.513	0.617

Mean ± SD, N=5 per sex/genotype and time point; Fold change: *Pkd1*$^{RC/RC}$ versus WT; ANOVA & Tukey's multiple comparison, P-values: *Pkd1*$^{RC/RC}$ versus wildtype *<0.05, **<0.01, ***<0.001, ****<0.0001; 3-months *Pkd1*$^{RC/RC}$ versus 6- or 9-months *Pkd1*$^{RC/RC}$ $^{\#}$<0.05, $^{\#\#}$<0.01. Normalized abundance/mg tissue: Raw abundance peak area of each metabolite normalized to (i) peak area of 12 deuterated internal standards, (ii) peak area of all metabolites across a individual sample, (iii) individual tissue weight.

Supplemental Table 2 | PKD parameters and tryptophan metabolite levels of *Pkd1*$^{RC/RC}$ mice with or without genetic *Ido1* loss separated by sex.

6 months	Male			Female		
	Pkd1 $^{RC/RC}$; *Ido1* $^{+/+}$	*Pkd1* $^{RC/RC}$; *Ido1* $^{-/-}$	p-value	*Pkd1* $^{RC/RC}$; *Ido1* $^{+/+}$	*Pkd1* $^{RC/RC}$; *Ido1* $^{-/-}$	p-value
%KW/BW	3.20±0.443	2.85±0.282	0.183	3.28±0.415	2.64±0.128	<u>0.0164</u>
Computed Cyst Vol. (µl)	291.4±87.5	190.8±28.1	<u>0.0401</u>	225.5±82.3	144.3±93.4	0.167
Cystic Index (%)	34.8±7.20	26.1±3.84	<u>0.0431</u>	29.9±7.07	21.3±9.08	0.111
Cyst Number/mm^2	14.8±2.94	12.9±2.17	0.274	17.0±2.08	14.9±1.59	0.110
Cyst Size (µm^2)	24,407±5,910	16,819±4,010	<u>0.0448</u>	17,822±3,991	13,980±4,917	0.184
Computed Fibrotic Vol. (µl)	20.5±13.3	22.5±12.5	0.812	30.8±17.5	28.0±25.4	0.829
Fibrotic Index (%)	2.46±1.52	3.02±1.50	0.576	4.04±1.78	3.98±2.72	0.965
BUN (mg/dl)	45.7±9.80	39.1±6.26	0.242	34.5±11.1	29.0±12.9	0.472
Tryptophan (ng/mg kidney tissue)	535.4±134.7	358.2±55.4	<u>0.0262</u>	322.7±121.3	268.3±96.6	0.464
Kynurenine (ng/mg kidney tissue)	2.04±0.378	0.860±0.261	<u>0.0004</u>	1.53±0.541	0.350±0.129	<u>0.0023</u>
Kynurenic acid (ng/mg kidney tissue)	11.5±4.16	6.88±1.30	<u>0.047</u>	6.47±2.68	5.40±1.85	0.500

Mean ± SD, N=4-7 per sex/genotype; unpaired t test, significant p-values are underlined

Supplemental Table 3 | PKD parameters and tryptophan metabolite levels of *Pkd1*$^{RC/RC}$ mice treated with or without 1-MT separated by sex.

	Male			Female		
Pkd1 $^{RC/RC}$	**Cntrl.**	**1-MT**	**p-value**	**Cntrl.**	**1-MT**	**p-value**
%KW/BW	2.73±0.259	2.08±0.405	0.0783	2.36±0.042	2.03±0.322	0.156
Computed Cyst Vol. (µl)	119.9±13.8	47.4±26.5	0.0136	63.1±10.0	26.0±9.30	0.0101
Cystic Index (%)	20.5±1.81	10.8±3.06	0.0090	16.6±2.20	8.50±1.78	0.0078
Cyst Number/mm^2	12.5±1.45	7.87±0.404	0.0058	13.9±0.819	8.80±1.41	0.0056
Cyst Size (µm^2)	16,582±2,128	13,640±3,029	0.241	12,086±1,466	9,552±606.7	0.0510
Computed Fibrotic Vol. (µl)	9.95±5.06	7.42±4.82	0.565	6.37±4.06	3.83±1.24	0.359
Fibrotic Index (%)	1.76±1.02	1.67±0.696	0.899	1.66±1.04	1.22±0.266	0.516
BUN (mg/dl)	54.4±22.6	44.6±11.5	0.542	41.8±8.82	40.4±8.86	0.857
Tryptophan (ng/mg kidney tissue)	600.1±21.9	283.4±185.8	0.0427	462.1±305.5	440.9±145.5	0.919
Kynurenine (ng/mg kidney tissue)	0.826±0.502	1.31±0.368	0.248	2.38±1.31	1.48±0.269	0.309
Kynurenic acid (ng/mg kidney tissue)	5.63±1.37	2.19±0.811	0.0201	4.85±1.20	2.48±0.569	0.0369

Mean ± SD, N=3 per sex/treatment; unpaired t test, significant p-values are underlined

Supplemental Table 4 | PKD parameters and tryptophan metabolite levels of juvenile induced *Pax8*^{rtTA};TetO-cre;*Pkd2*$^{flx/flx}$ mice treated with or without 1-MT separated by sex.

	Male			Female		
Pkd1 $^{RC/RC}$	**Cntrl.**	**1-MT**	**p-value**	**Cntrl.**	**1-MT**	**p-value**
%KW/BW	9.68±2.19	6.77±2.52	0.0744	7.14±0.814	5.28±0.580	<u>0.0051</u>
Computed Cyst Vol. (µl)	385.8±118.4	177.3±95.3	<u>0.0101</u>	245.0±34.4	132.2±41.0	<u>0.0032</u>
Cystic Index (%)	39.6±5.65	26.4±7.27	<u>0.0091</u>	35.8±4.86	25.9±6.13	<u>0.0344</u>
Cyst Number/mm^2	25.7±5.40	18.5±3.63	<u>0.0271</u>	35.2±18.6	26.1±14.5	0.438
Cyst Size (µm^2)	17,255±2,661	14,450±2,518	0.106	12,856±5,926	11,552±3,309	0.686
Computed Fibrotic Vol. (µl)	27.4±9.22	11.4±6.52	<u>0.0084</u>	13.3±3.62	7.04±2.23	<u>0.0153</u>
Fibrotic Index (%)	2.80±0.449	1.72±0.574	<u>0.0077</u>	1.90±0.296	1.37±0.379	0.0621
BUN (mg/dl)	83.2±20.5	75.0±13.5	0.512	81.2±17.2	66.1±0.02	0.171
Tryptophan (ng/mg kidney tissue)	260.2±54.55	368.2±173.5	0.347	317.4±174.9	223.7±147.8	0.444
Kynurenine (ng/mg kidney tissue)	1.27±0.426	1.04±0.473	0.525	1.05±0.182	1.27±0.481	0.426
Kynurenic acid (ng/mg kidney tissue)	12.2±8.21	7.64±3.24	0.294	7.34±5.34	3.77±1.91	0.251

Mean ± SD, N=3-6 per sex/treatment; unpaired t test, significant p-values are underlined

References

1. Research IoLA. *Guide for the Care and Use of Laboratory Animals.* Washington (DC): National Academies Press; 2011.
2. Arroyo J, Escobar-Zarate D, Wells HH, Constans MM, Thao K, Smith JM, et al. The genetic background significantly impacts the severity of kidney cystic disease in the Pkd1(RC/RC) mouse model of autosomal dominant polycystic kidney disease. *Kidney Int.* 2021.
3. Hopp K, Ward CJ, Hommerding CJ, Nasr SH, Tuan HF, Gainullin VG, et al. Functional polycystin-1 dosage governs autosomal dominant polycystic kidney disease severity. *J Clin Invest.* 2012;122(11):4257-73.
4. Kleczko EK, Marsh KH, Tyler LC, Furgeson SB, Bullock BL, Altmann CJ, et al. CD8(+) T cells modulate autosomal dominant polycystic kidney disease progression. *Kidney Int.* 2018;94(6):1127-40.
5. Garcia-Gonzalez MA, Outeda P, Zhou Q, Zhou F, Menezes LF, Qian F, et al. Pkd1 and Pkd2 are required for normal placental development. *PLoS One.* 2010;5(9).
6. Traykova-Brauch M, Schonig K, Greiner O, Miloud T, Jauch A, Bode M, et al. An efficient and versatile system for acute and chronic modulation of renal tubular function in transgenic mice. *Nat Med.* 2008;14(9):979-84.
7. Perl AK, Wert SE, Nagy A, Lobe CG, and Whitsett JA. Early restriction of peripheral and proximal cell lineages during formation of the lung. *Proc Natl Acad Sci U S A.* 2002;99(16):10482-7.
8. Hou DY, Muller AJ, Sharma MD, DuHadaway J, Banerjee T, Johnson M, et al. Inhibition of indoleamine 2,3-dioxygenase in dendritic cells by stereoisomers of 1-methyl-tryptophan correlates with antitumor responses. *Cancer Res.* 2007;67(2):792-801.

9. Pigott E, DuHadaway JB, Muller AJ, Gilmour S, Prendergast GC, and Mandik-Nayak L. 1-Methyl-tryptophan synergizes with methotrexate to alleviate arthritis in a mouse model of arthritis. *Autoimmunity.* 2014;47(6):409-18.

10. Zeng J, Cai S, Yi Y, He Y, Wang Z, Jiang G, et al. Prevention of spontaneous tumor development in a ret transgenic mouse model by ret peptide vaccination with indoleamine 2,3-dioxygenase inhibitor 1-methyl tryptophan. *Cancer Res.* 2009;69(9):3963-70.

11. Taher YA, Piavaux BJ, Gras R, van Esch BC, Hofman GA, Bloksma N, et al. Indoleamine 2,3-dioxygenase-dependent tryptophan metabolites contribute to tolerance induction during allergen immunotherapy in a mouse model. *J Allergy Clin Immunol.* 2008;121(4):983-91 e2.

12. Chen X, Liu L, Yang P, Wu C, Jin H, Xing L, et al. Indoleamine 2,3-dioxygenase (IDO) is involved in promoting the development of anterior chamber-associated immune deviation. *Immunol Lett.* 2006;107(2):140-7.

13. Sakurai K, Zou JP, Tschetter JR, Ward JM, and Shearer GM. Effect of indoleamine 2,3-dioxygenase on induction of experimental autoimmune encephalomyelitis. *J Neuroimmunol.* 2002;129(1-2):186-96.

14. Gainullin VG, Hopp K, Ward CJ, Hommerding CJ, and Harris PC. Polycystin-1 maturation requires polycystin-2 in a dose-dependent manner. *J Clin Invest.* 2015;125(2):607-20.

15. Yuan M, Breitkopf SB, Yang X, and Asara JM. A positive/negative ion-switching, targeted mass spectrometry-based metabolomics platform for bodily fluids, cells, and fresh and fixed tissue. *Nat Protoc.* 2012;7(5):872-81.

16. Baliga MM, Klawitter J, Christians U, Hopp K, Chonchol M, Gitomer BY, et al. Metabolic profiling in children and young adults with autosomal dominant polycystic kidney disease. *Sci Rep.* 2021;11(1):6629.

17. Zhu W, Stevens AP, Dettmer K, Gottfried E, Hoves S, Kreutz M, et al. Quantitative profiling of tryptophan metabolites in serum, urine, and cell culture supernatants by liquid chromatography-tandem mass spectrometry. *Anal Bioanal Chem.* 2011;401(10):3249-61.

18. Chong J, Soufan O, Li C, Caraus I, Li S, Bourque G, et al. MetaboAnalyst 4.0: towards more transparent and integrative metabolomics analysis. *Nucleic Acids Research.* 2018;46(W1):W486-W94.
